# Epigenetic Reprogramming by Decitabine in Triple-Negative Breast Cancer: Mechanisms, Immune Modulation, and Therapeutic Synergy

**DOI:** 10.3390/cancers17182953

**Published:** 2025-09-09

**Authors:** Fathima Raahima Riyas Mohamed, Safiah Aldubaisi, Arshiya Akbar, Mohammad Imran Khan, Ahmed Yaqinuddin

**Affiliations:** 1 College of Medicine, Alfaisal University, Riyadh 50927, Saudi Arabia; frmohamed@alfaisal.edu (F.R.R.M.); saldubaisi@alfaisal.edu (S.A.); arshiyaakbar2019@gmail.com (A.A.); 2 King Faisal Specialist Hospital and Research Center, Jeddah 40047, Saudi Arabia; mikhan@kfshrc.edu.sa

**Keywords:** triple-negative breast cancer, TNBC, decitabine, DNA methyltransferase inhibitor, 5-aza-2′-deoxycytidine, DNMTi

## Abstract

**Background/Objectives**: Triple-negative breast cancer (TNBC) is an aggressive subtype lacking ER, PR, and HER2 expression, with limited targeted therapies and poor outcomes. Epigenetic dysregulation, particularly aberrant DNA methylation, is a key driver. Decitabine, a DNA methyltransferase inhibitor (DNMTi), shows promise by reactivating silenced tumor suppressor genes and modulating immune responses. This systematic review evaluates preclinical and clinical evidence on decitabine’s efficacy, mechanisms, and translational potential in TNBC. **Methods**: A PRISMA-2020 compliant search of PubMed, EBSCO, Web of Science, and Semantic Scholar was conducted up to April 2025. Included studies assessed decitabine alone or in combination in TNBC preclinical or clinical settings. Risk of bias was assessed using QUIPS and RoB 2.0 tools. **Results**: Twenty-five studies were included. In vitro, decitabine-induced growth inhibition, apoptosis, and re-expression of silenced genes (such as *BRCA1* and *CDH1*). In vivo, it reduced tumor burden and enhanced anti-tumor immunity through MHC-I, PD-L1, and STING pathway upregulation. Synergy was noted with anti-PD-1, HDAC inhibitors, and chemotherapy. Resistance mechanisms included persistent DNMT activity, low DCK, and miRNA-driven escape (miR-155–TSPAN5). **Conclusions**: Decitabine demonstrates strong preclinical and early clinical potential in TNBC via epigenetic reprogramming and immune activation. Future strategies should focus on biomarker-based selection and resistance mitigation.

## Simple Summary

Decitabine, a DNA methyltransferase inhibitor, shows potential in treating triple-negative breast cancer (TNBC) by reactivating tumor suppressor genes and enhancing immune responses. Preclinical studies demonstrate its ability to reduce tumor growth, induce apoptosis, and improve sensitivity to chemotherapy, immunotherapy, and hormonal agents. Notably, combination therapies such as decitabine with anti-PD-1, HDAC inhibitors, or paclitaxel converting immune “cold” tumors into “hot” ones, improves outcomes. Resistance may arise from persistent DNMT expression, low DCK activity, and miRNA-regulated stemness pathways, suggesting a need for biomarker-based patient selection. A Phase II trial combining decitabine with pembrolizumab achieved a 40.7% pathologic complete response in early stage TNBC, supporting its promise in combinatorial regimens.

## 1. Introduction

Triple-negative breast cancer (TNBC) is a clinically and molecularly distinct subtype of breast cancer defined by the absence of estrogen receptor (ER), progesterone receptor (PR), and human epidermal growth factor receptor 2 (HER2) expression [1,2]. It accounts for approximately 15–20% of all breast cancer cases and is associated with disproportionately poor clinical outcomes [3,4]. TNBC typically affects younger women and is more prevalent in patients of African and Hispanic descent [5,6,7]. It is known for its high histological grade, rapid proliferation, and early metastatic potential, particularly to visceral organs and the brain [8,9]. Unlike hormone receptor-positive or HER2-amplified breast cancers, TNBC lacks actionable molecular targets, leaving systemic chemotherapy as the primary therapeutic option [10,11,12]. Despite initial responsiveness, recurrence is common (approximately around 40%) and often occurs within the first three years of diagnosis [13,14]. Survival outcomes also vary significantly by stage: the 5-year relative survival rate is 91–92% for localized TNBC, 65–67% for regional disease, and only 11–15% for distant metastases, with an overall 5-year survival across all stages of approximately 77–78%. These figures underscore the aggressive nature of the disease and the urgent need for novel therapeutic strategies [13,14].

One of the most pressing challenges in TNBC management is its heterogeneity and lack of effective targeted therapies [15,16]. Molecular profiling has revealed that TNBC encompasses multiple subtypes with distinct transcriptomic signatures, including basal-like [17], mesenchymal [18], immunomodulatory [19], and luminal androgen receptor-positive variants [20]. However, these molecular distinctions have yet to translate into widely adopted therapeutic approaches. Furthermore, TNBC is often enriched with features such as epithelial-to-mesenchymal transition (EMT) [21], cancer stem cell-like phenotypes [22], immune evasion [23,24], and chemotherapy resistance [25], complicating treatment responses and fueling relapse.

In recent years, attention has turned toward epigenetic dysregulation as a critical driver of TNBC tumorigenesis and progression [26,27]. Epigenetic alterations, heritable changes in gene expression without changes to the underlying DNA sequence, include DNA methylation, histone modification, and non-coding RNA regulation [28,29]. Among these, DNA methylation has been particularly implicated in TNBC pathobiology [30,31]. Aberrant hypermethylation of CpG islands in promoter regions of tumor suppressor genes such as BRCA1, RASSF1A, CDH1, and p16^INK4a leads to their transcriptional silencing and contributes to unchecked cellular proliferation, impaired DNA repair, and immune escape [32,33]. Conversely, global DNA hypomethylation is linked to genomic instability and the activation of oncogenes [34,35,36]. Importantly, epigenetic alterations are reversible, rendering them promising therapeutic targets in cancers characterized by such dysregulation [37].

Decitabine (5-aza-2′-deoxycytidine) is a cytosine analog and DNA methyltransferase inhibitor (DNMTi) approved for the treatment of hematological malignancies such as myelodysplastic syndromes (MDS) and acute myeloid leukemia (AML) [38,39]. Its mechanism of action is grounded in the covalent trapping and degradation of DNMT1 [40], the enzyme responsible for maintaining DNA methylation patterns during replication [41]. Upon incorporation into replicating DNA, decitabine induces passive demethylation by depleting DNMT1 levels, leading to the reactivation of silenced tumor suppressor genes and epigenetic reprogramming of cancer cells [39,40,42]. Additionally, decitabine has been shown to influence the tumor microenvironment by enhancing antigen presentation, promoting immune cell infiltration, and synergizing with the immune checkpoint blockade, suggesting potential immunomodulatory effects [43,44].

Preclinical studies in TNBC models have demonstrated that decitabine can reverse key features of the malignant phenotype, including stemness, mesenchymal traits, and chemoresistance while restoring immune visibility and sensitizing tumors to immune checkpoint inhibitors and cytotoxic agents [26,45,46,47]. These findings have sparked interest in repositioning decitabine for solid tumors, including TNBC, despite historical skepticism about the efficacy of hypomethylating agents in such settings [48,49]. Early-phase clinical trials exploring low-dose decitabine regimens—designed to minimize cytotoxicity and maximize epigenetic reprogramming—have begun to emerge, evaluating its role as monotherapy or in combination with chemotherapy, PARP inhibitors, and immunotherapy [50,51,52].

Given TNBC’s poor prognosis, resistance to conventional therapies, and clear evidence of epigenetic disruption, a systematic appraisal of decitabine’s role in this context is both timely and necessary. Although promising data exist, the landscape remains fragmented, with varied dosing schedules, biomarkers, and outcome measures reported across studies. To date, no comprehensive review has integrated the mechanistic, preclinical, and clinical evidence supporting the use of decitabine in TNBC.

This systematic review therefore aims to synthesize and critically analyze the current body of preclinical and clinical literature evaluating decitabine in TNBC. Specifically, we explore the following:

- The mechanistic rationale for its use based on epigenetic alterations in TNBC;
- Evidence of its efficacy and synergistic potential in preclinical models;
- Clinical outcomes, safety profiles, and combinatorial strategies being tested;
- Existing challenges and gaps in translating epigenetic therapy into clinical benefit;
- By consolidating this knowledge, we aim to inform future translational research and clinical trial design, with the goal of expanding therapeutic options for patients with this formidable breast cancer subtype.

## 2. Materials and Methods

This systematic review adheres to the PRISMA-2020 (Preferred Reporting Items for Systematic Reviews and Meta-Analyses) [53] and AMSTAR (assessing the methodological quality of systematic reviews) [54] guidelines to ensure methodological rigor and transparency (refer to Tables S1 and S2). The scope and objectives of the review are structured based on the PICOS framework (Participants, Interventions, Comparisons, Outcomes, and Study Designs), as outlined in Table 1 [55].

### 2.1. Literature Search

The search spanned multiple major databases—PubMed, EBSCO, Web of Science, and Semantic Scholar. To ensure retrieval accuracy, the search strategy included a combination of keywords and Medical Subject Headings (MeSH) terms, utilizing terms such as (Decitabine OR Azacitidine OR DNMT inhibitor OR 5-aza-2′-deoxycytidine OR DNA hypomethylating agents) AND (TNBC OR Triple-negative breast cancer). Boolean operators (AND/OR) were applied to structure the syntax effectively, targeting studies specifically on the role of decitabine in the treatment of TNBC. This search was conducted across titles, abstracts, and keywords and concluded on 26 April 2025. All citations were imported into Rayyan software (version 1.5.3) for subsequent processing, including duplicate removal and initial screening [56].

### 2.2. Inclusion and Exclusion Criteria

The inclusion criteria for this systematic review were defined to comprehensively capture studies that directly investigate the therapeutic role of decitabine in triple-negative breast cancer (TNBC) across both clinical and preclinical contexts. Eligible studies were those involving human participants diagnosed with TNBC or employing preclinical models such as in vitro TNBC cell lines (such as MDA-MB-231, HCC1937, and BT-549) or in vivo animal models (such as murine xenografts or syngeneic mouse models) derived from TNBC tumors. The intervention of interest was the administration of decitabine, whether as a single agent or in combination with other therapies, such as chemotherapeutics, immune checkpoint inhibitors, or PARP inhibitors. For comparison, studies were included if they had a control group receiving either standard-of-care treatments (such as anthracyclines or taxanes), immunotherapy, or placebo/vehicle controls. Eligible studies had to report on at least one relevant outcome related to tumor response, such as overall response rate (ORR), progression-free survival (PFS), overall survival (OS), disease-free survival (DFS), or biomarker responses, particularly those reflecting epigenetic modulation (like DNA methylation levels and DNMT1 expression) or immune alterations (like T-cell infiltration and cytokine profiles). Studies were also included if they addressed safety profiles and treatment-related adverse events, given the known hematologic toxicity of DNMT inhibitors. Finally, the review accepted a broad range of study designs, including randomized controlled trials (RCTs), non-randomized clinical trials, cohort studies, and preclinical in vivo and in vitro experimental studies, to ensure a robust synthesis of evidence across different stages of translational research.

Studies were excluded if they did not specifically focus on triple-negative breast cancer (TNBC), for instance, those investigating hormone receptor-positive or HER2-positive breast cancer subtypes were omitted due to their differing biology and treatment responses. Additionally, studies in which decitabine was not the primary therapeutic intervention or where it was only mentioned peripherally were excluded to maintain a clear focus on the agent of interest. The review also excluded non-original research such as review articles, editorials, opinion pieces, conference abstracts, case reports, and letters, as these do not provide primary data suitable for extraction or quantitative synthesis. Furthermore, studies were excluded if they lacked full-text availability, were not published in English, or failed to report relevant outcome data, such as therapeutic response rates, survival outcomes, molecular or immune biomarkers, or evidence of epigenetic modulation. These exclusions were necessary to uphold the scientific quality and interpretability of the final body of evidence.

### 2.3. Literature Screening

The initial screening process was systematic, beginning with title screening followed by abstract screening. Each article’s title and abstract were carefully evaluated against predefined inclusion and exclusion criteria. After this initial stage, a second round of evaluation involved a detailed assessment of the full-text articles. This stage was critical for refining the selection based on the same criteria, with particular emphasis on each study’s rigor and relevance to the research objectives. Full-text articles were meticulously reviewed to ensure they not only addressed the role of decitabine in the treatment of triple-negative breast cancers, but also provided adequate scientific detail on the immune responses, anti-tumor immunity, and epigenetic modulations under investigation. Discrepancies in study inclusion were resolved through consensus discussion between reviewers. This rigorous three-step screening process ensured that the final selection of studies would form a robust and relevant dataset for understanding the treatment of TNBC using decitabine.

### 2.4. Data Extraction

Data were extracted from each included study using a structured Microsoft Excel form to ensure a thorough capture of essential information. The form collected study identification details, such as the first author, publication year, study title, and study design. Other characteristics were also documented, including type of cell line or animal model used, source of cells or tumors, and total sample size. Intervention-specific variables were recorded, detailing the type of intervention, treatment duration, dosage (mg/m^2^ or µM), and mechanism of action. Outcome data were extracted as well, including biomarkers or molecular targets, tumor progression or growth, survival (progression-free survival and overall survival), tumor histopathology, immune metrics, and epigenetic modulation.

### 2.5. Risk of Bias Assessment

To ensure a rigorous and transparent assessment of methodological quality and risk of bias across the diverse range of study designs included in this systematic review, two validated tools were employed based on the nature of the study. For observational studies, the Quality in Prognosis Studies (QUIPS) tool was used, which is specifically designed to assess the risk of bias in studies investigating prognostic factors or associations in non-randomized settings. The QUIPS tool evaluates six key domains: study participation, assessing whether the study population is representative of the target population; study attrition, examining the completeness of follow-up and reasons for loss to follow-up; prognostic factor measurement, ensuring accurate and consistent assessment of variables such as biomarker expression or molecular response; outcome measurement, focusing on the reliability and objectivity of endpoints like overall survival or progression-free survival; study confounding, which assesses whether potential confounding variables were adequately identified and adjusted for; and statistical analysis and reporting, evaluating the appropriateness and transparency of statistical methods used. Each domain was rated as having a low (***), moderate (**), or high risk of bias (*), and a summary judgment was made for each study [57].

For randomized controlled trials (RCTs) included in the review, risk of bias was assessed using the Cochrane Risk of Bias 2.0 (RoB 2) tool, the gold standard for evaluating the internal validity of clinical trials. The RoB 2 tool assesses five specific domains: bias arising from the randomization process, which examines sequence generation and allocation concealment; bias due to deviations from intended interventions, assessing whether patients remained adherent to their assigned groups and whether analyses were conducted according to the intention-to-treat principle; bias due to missing outcome data, evaluating the extent of loss to follow-up and whether it could bias the results; bias in measurement of the outcome, focusing on whether outcome assessors were blinded and whether outcome definitions were applied consistently; and bias in the selection of the reported result, determining whether pre-specified outcomes were selectively reported. Each domain was judged as “low risk,” “some concerns,” or “high risk” of bias, and an overall risk of bias was assigned to each trial [58].

All studies were independently assessed by two reviewers with expertise in systematic review methodology. Discrepancies in scoring were resolved through detailed discussion and consensus. This multi-tiered, domain-specific approach to risk of bias evaluation ensured that both clinical and observational evidence were scrutinized with appropriate methodological tools, enhancing the reliability and validity of the review’s conclusions.

## 3. Results

### 3.1. Study Selection

The initial screening of identified studies began with a comprehensive review of their titles and abstracts to assess relevance based on the predefined PICOS criteria established for this systematic review. This multi-database search yielded a total of 896 records, which were systematically imported into Rayyan software to facilitate an organized and efficient screening process. Upon import, Rayyan identified and removed 30 duplicate entries, leaving 866 unique records available for evaluation.

The first phase of the screening process involved a meticulous examination of the titles and abstracts against the inclusion and exclusion criteria. Title screening resulted in the exclusion of 770 records that were either irrelevant to TNBC research or lacked focus on treatment with Decitabine. Subsequently, 96 abstracts were selected for further eligibility assessment.

During this secondary phase, each article underwent a detailed evaluation to ensure adherence to the inclusion criteria. A total of 74 full-texts were assessed for eligibility, out of which 47 studies were excluded for various reasons: two studies had no access to their full texts, 18 studies had insufficient data on TNBC and decitabine, 26 studies did not specifically focus on TNBC, one terminated the clinical trial, and two studies had the wrong study design. Exclusions were clearly documented to maintain transparency in the selection process.

**Figure 1:**
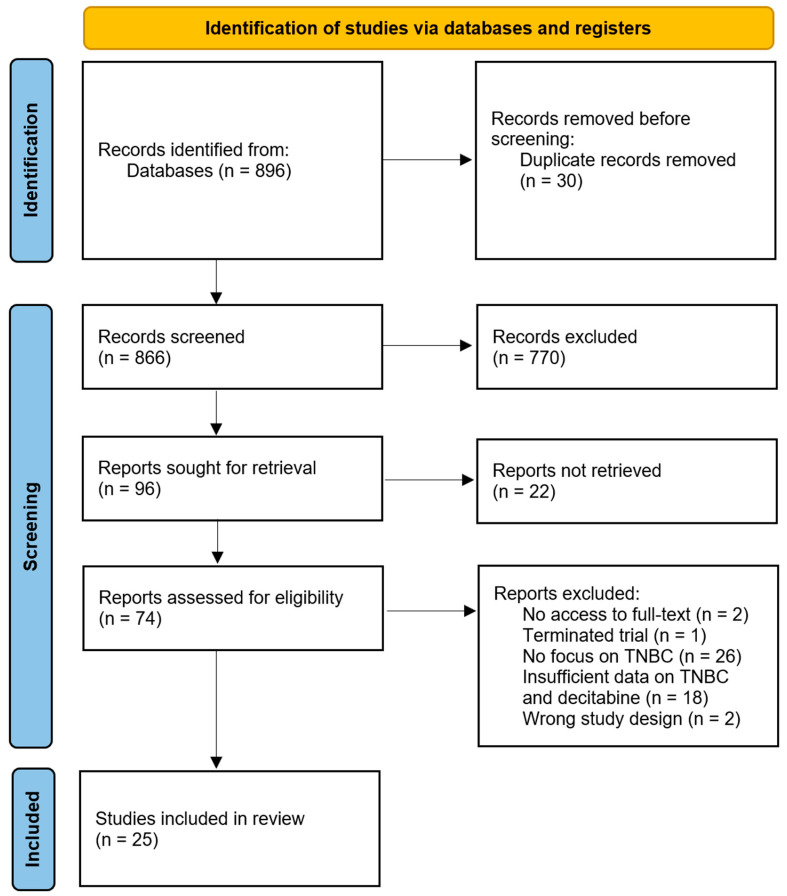
Preferred Reporting Items for Systematic Reviews and Meta-Analyses (PRISMA) diagram demonstrating search strategy.

Finally, 25 studies were deemed eligible and included in the qualitative synthesis. These studies spanned various experimental designs, including preclinical models and mechanistic investigations, to provide a comprehensive analysis of the role of decitabine in the treatment of TNBC. This rigorous multi-stage review process, in line with PRISMA guidelines, underscored a systematic approach to identifying high-quality evidence pertinent to the review objectives, as depicted in Figure 1.

### 3.2. Risk of Bias Assessment

**Figure 2:**
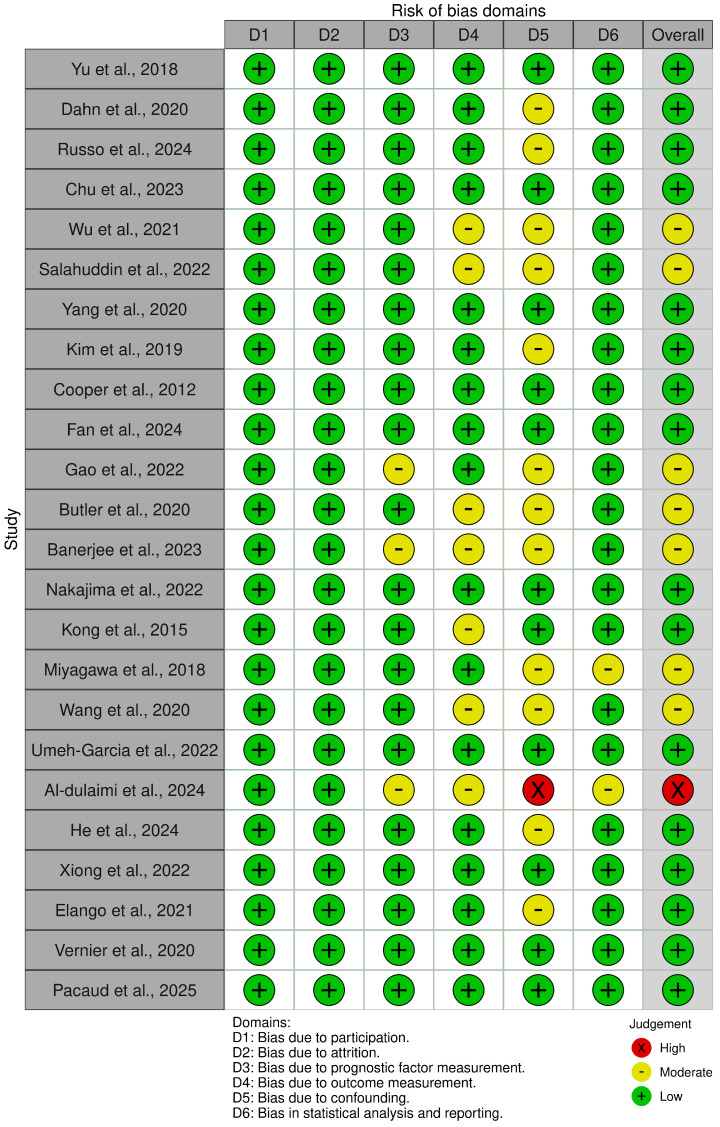
Traffic light plot demonstrating the risk of bias assessment using the Quality in Prognosis Studies (QUIPS) tool [45,47,59,60,61,62,63,64,65,66,67,68,69,70,71,72,73,74,75,76,77,78,79,80].

**Figure 3:**
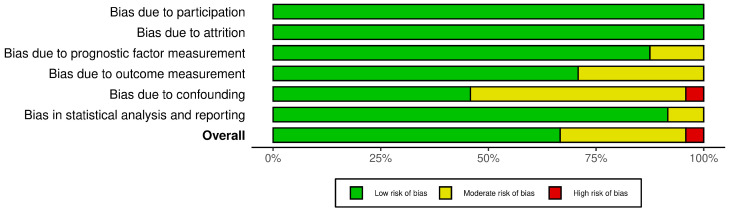
Summary plot demonstrating the risk of bias assessment using the Quality in Prognosis Studies (QUIPS) tool.

The risk of bias in the preclinical studies was conducted using the QUIPS risk of bias assessment tool (refer to Table S3) [57]. As summarized in Figure 2 and Figure 3, the majority of studies (n = 16) were assessed as having a low overall risk of bias, reflecting well-characterized TNBC models, complete datasets with minimal attrition, appropriate use of outcome measures such as tumor volume, gene expression, or immune markers, and generally robust statistical reporting [45,47,59,60,61,62,63,64,65,66,67,68,69,70,71,72]. A subset of seven studies were judged to have a moderate risk of bias, most commonly due to issues in the domains of study confounding and statistical analysis [65,73,74,75,76,77,78]. These studies often lacked appropriate mechanistic controls (such as rescue or knockout experiments) or did not fully disentangle the effects of multi-agent regimens (for instance, DAC + HDACi + chemotherapy) on observed outcomes. In several cases, quantitative findings were reported without detailed information on biological replicates, significance thresholds, or confidence intervals, limiting interpretability. Additionally, in studies where immune reprogramming was inferred solely from gene expression data without functional validation (such as T-cell killing assays), the outcome measurement domain was downgraded accordingly. High-quality studies such as those by Yu et al., 2018, Dahn et al., 2020, Umeh-Garcia et al., 2020 and Russo et al., 2024 scored low across all domains due to use of both mechanistic models and confirmatory functional assays, appropriate control groups, and transparent, reproducible statistical frameworks [45,47,59,79]. In contrast, only one study (Al-Dulaimi et al., 2024) was classified as having a high risk of bias, due to insufficient methodological detail, lack of protein-level or functional validation, and minimal reporting of statistical procedures [80]. 

**Figure 4:**
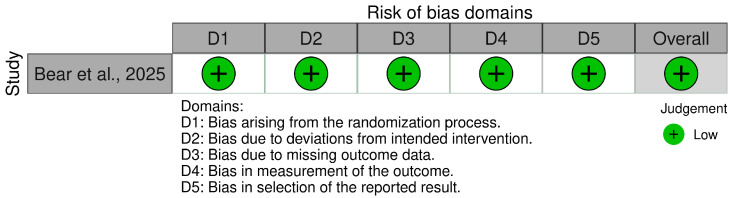
Traffic light plot demonstrating the risk of bias assessment using the Cochrane ROB-2 tool [52].

The risk of bias in the clinical trial conducted by Bear et al., 2025 [52] was evaluated using the Cochrane Risk of Bias 2.0 (RoB 2) tool (refer to Table S4) [58], and the results are summarized in Figure 4. Due to the single-arm, non-randomized design, the study was rated as having a high risk of bias in Domain 1 (bias arising from the randomization process). Domains 2, 3, and 4, each assessing deviations from intended interventions, missing outcome data, and measurement of the outcomes, were judged to have a low risk of bias, reflecting adherence to the treatment protocol, minimal attrition, and use of objective, standardized methods for outcome measurement. Domain 5 (bias in selection of the reported result) was rated as having some concerns, as the absence of a referenced pre-specified analysis plan limited the ability to fully exclude selective reporting. Based on these domain-level judgments, the study was assigned an overall risk of bias of “moderate” [52].

### 3.3. Study Characteristics

The 25 included studies focused on triple-negative breast cancer (TNBC) models, evaluating the role of decitabine (DAC) as a hypomethylating agent either as monotherapy or in combination with immunotherapy, chemotherapy, or epigenetic modulators. Experimental platforms were diverse: human TNBC cell lines (such as MDA-MB-231, BT-549, Hs578T) for mechanistic exploration, syngeneic mouse models (such as 4T1, 66cl4) for immunological profiling, and PDX systems to evaluate therapeutic durability and biomarker stratification. A small subset of studies also examined non-TNBC subtypes for comparative epigenetic analyses.

Geographic representation was broad, with studies conducted across the United States, China, Finland, Egypt, Canada, Taiwan, and Italy, reflecting the global interest in epigenetic therapy for breast cancer. Refer to Table S5 for detailed characteristics of all the included studies.

### 3.4. Anti-Tumor Efficacy of Decitabine

Decitabine (DAC), as a DNA hypomethylating agent, demonstrated consistent anti-tumor efficacy across a wide range of triple-negative breast cancer (TNBC) models, including in vitro cell lines, xenograft models, syngeneic murine models, and patient-derived xenografts (PDXs).

#### 3.4.1. In Vitro Evidence of Cytotoxicity and Clonogenic Suppression

In vitro studies consistently demonstrated that decitabine (DAC) exerts potent antiproliferative effects on TNBC cell lines through dose- and time-dependent reductions in cell viability, colony formation, and proliferation (summarized in Table 2). Dahn et al., 2020 reported that DAC significantly inhibited colony-forming efficiency in MDA-MB-231, MDA-MB-468, and SUM159 cells, an effect mechanistically linked to the reactivation of silenced tumor suppressor genes such as BRCA1 and CDH1 [47]. Kim et al., 2019 further demonstrated that DAC, when co-administered with the HDAC inhibitor panobinostat via lipid nanoemulsions (LNEs), synergistically reduced TNBC cell viability by 60–90%, while sparing normal mammary epithelial cells, highlighting both efficacy and selectivity [62]. Cooper et al., 2012 noted that DAC alone produced modest antiproliferative effects; however, when combined with romidepsin, a marked increase (10–21%) in apoptosis was observed, confirmed by elevated levels of cleaved PARP and caspase-3, underscoring the potential of DAC as a sensitizer in epigenetic combination strategies [63]. Collectively, these in vitro findings affirm DAC’s cytotoxic efficacy and suggest a role for its integration into multi-modal therapeutic regimens for TNBC.

#### 3.4.2. Tumor Volume Reduction in Murine Models

Studies utilizing in vivo murine models, including both xenografts and syngeneic systems, consistently demonstrated tumor growth suppression with decitabine (DAC), often in combination with other agents (refer to Table 3). For example, Russo et al., 2024 reports that DAC monotherapy moderately delayed tumor progression; however, when combined with PeptiCRAd, the treatment significantly inhibited tumor volume increase by day 28 post-inoculation, indicating a synergistic effect through enhanced immune activation [59]. Wu et al., 2021 states that DAC monotherapy caused a marked reduction in tumor growth compared to vehicle controls [73]. Furthermore, the combination of DAC with anti-PD-1 therapy dramatically amplified tumor suppression effects and prolonged survival [73]. Among all treatment arms in the study conducted by Gao et al., 2022, the combination nanoparticle group showed the most significant tumor volume reduction, and longest survival [65]. Tumor weights were significantly reduced, and apoptosis (TUNEL staining) and necrosis were markedly increased [65]. While tumor volume endpoints were not directly measured due to early sacrifice in the study conducted by Banerjee et al., 2023, histological analysis showed extensive necrosis and apoptosis in the combination therapy group [76]. This indicates that DAC primed the tumor microenvironment to respond more robustly to cytotoxic and immune-mediated damage, even in the absence of repeated DAC dosing.

#### 3.4.3. Patient-Derived Xenograft (PDX) Models and Clinical Translation

Patient-derived xenograft (PDX) models offer high translational relevance due to their ability to retain the histopathological, molecular, and therapeutic response profiles of original patient tumors.

Yu et al., 2018 developed 15 distinct TNBC PDX models from pre- and post-chemotherapy clinical specimens [45]. These were further categorized into DAC-sensitive and DAC-resistant subgroups based on both in vivo tumor shrinkage and in vitro organoid viability following DAC exposure (100 nM for 7 days). Six PDX models were tested in vivo: MCD-01, -03, and -11 were sensitive, whereas MCD-02, -04, and -05 were resistant. DAC treatment (5 mg/kg, intraperitoneally, 3×/week) resulted in durable tumor suppression in the sensitive PDXs with minimal regrowth during washout periods. In contrast, resistant PDXs exhibited early tumor rebound, highlighting inter-patient heterogeneity in DAC response. Mechanistically, DAC induced post-translational degradation of DNMT1, DNMT3A, and DNMT3B via TRAF6-mediated ubiquitination. This was independent of mRNA changes, suggesting epigenetic reprogramming at the protein level. TRAF6 knockout restored DNMT protein levels and conferred DAC resistance. Furthermore, DAC re-sensitized resistant organoids to paclitaxel, supporting a rationale for combination epigenetic–chemotherapy approaches [45].

In a multicenter Phase II trial, Bear et al., 2025 evaluated DAC (15 mg/m^2^ IV ×4 days) in combination with pembrolizumab (200 mg IV on Days 8 and 22) as a neoadjuvant priming strategy in early-stage TNBC patients. A total of 28 TNBC patients were enrolled across cohorts A and A2 [52]. Key immunological changes after the DAC + pembrolizumab “window” treatment included the following:

- Stromal TILs (sTILs): increased by 6.1% (*p* = 0.008);
- PD-L1 H-score: increased by 51.1% (*p* = 0.012);
- Monocytic MDSCs: decreased by 59% in blood (*p* < 0.01).

These changes were linked to increased immune infiltration and improved immune priming. The pathologic complete response (pCR) rate among TNBC patients was 40.7% (11/27), with only two recurrences at one year. Histologically, some post-treatment biopsies demonstrated lymphocyte-predominant breast cancer (LPBC) features, suggesting a shift from “cold” to “hot” immune phenotypes induced by epigenetic priming [52].

### 3.5. Immune Landscape Modulation

#### 3.5.1. Antigen Presentation and T-Cell Recruitment

Decitabine (DAC) enhances tumor immunogenicity by upregulating antigen presentation machinery and modulating T-cell infiltration and phenotype. This effect is primarily mediated through epigenetic reprogramming of key immune regulatory pathways (Table 4). According to the study conducted by Russo et al., 2024, treatment with low-dose DAC (0.5 mg/kg/day, i.p., ×5 days) significantly increased expression of MHC class I molecules and PD-L1 on tumor cells [59]. This antigen-presentation reactivation was attributed to DAC-induced hypomethylation of promoter regions involved in immune sensing, particularly ERV elements that function as immunogenic “danger signals” upon demethylation. The effect was most pronounced when combined with PeptiCRAd, which recruited and reprogrammed tumor-infiltrating lymphocytes (TILs), shifting CD8^+^ cells from a perivascular localization (control) to a more intratumoral distribution. The proportion of exhausted CD8^+^ T cells (PD-1^+^TIM-3^+^) dropped from 45% in control to 20% in the combination group, demonstrating reduced immune exhaustion [59].

Wu et al., 2021 extended this concept in MYC-driven TNBC models (4T1 and 66cl4), where overexpression of MYC was shown to suppress STING (Stimulator of Interferon Genes) expression via DNMT1-mediated promoter hypermethylation [73]. Treatment with DAC (0.8 mg/kg/day, i.p., for 3–5 weeks) demethylated the STING promoter, restoring its transcriptional activity. Reactivation of STING led to the upregulation of type I interferons (notably IFN-β), transcriptional induction of ISGs including MHC-I genes and chemokines CCL5 and CXCL10, and re-expression of PD-L1, a known interferon-response element. This led to elevated type I interferon signaling (↑ IFN-β, CCL5, CXCL10), enhanced CD8^+^ infiltration, and increased cytotoxicity (↑ granzyme B^+^ cells) [73].

#### 3.5.2. Combination with Immune Checkpoint Inhibitors

One of the most clinically impactful applications of decitabine (DAC) in triple-negative breast cancer (TNBC) involves its ability to enhance the efficacy of immune checkpoint inhibitors (ICIs), particularly anti-PD-1 therapies (Table 5). Through epigenetic reprogramming, DAC reactivates antigen presentation pathways and restores innate immune sensing, thereby converting immunologically “cold” TNBC tumors into “hot” tumors capable of responding to T cell-based therapies. As per Wu et al., 2021, DAC alone led to modest tumor control, but DAC + anti-PD-1 therapy produced synergistic tumor regression, prolonged survival, and enhanced granzyme B^+^ CD8^+^ cytotoxic T-cell activation [73]. In STING-knockout tumors, the synergy was abolished, confirming that STING-mediated innate immune activation is a prerequisite for PD-1 responsiveness in MYC-driven TNBC [73]. In the Bear et al., 2025 clinical trial, treatment with decitabine and pembrolizumab resulted in significant modulation of the tumor immune microenvironment. Notably, PD-L1 H-scores increased by 51.1% (*p* = 0.012), while stromal tumor-infiltrating lymphocytes (sTILs) rose by 6.1% (*p* = 0.008) following the priming phase [52]. Concurrently, there was a 59% reduction in circulating monocytic myeloid-derived suppressor cells (M-MDSCs), indicating a decrease in systemic immunosuppression. Histologically, three out of eleven evaluable patients developed a lymphocyte-predominant breast cancer (LPBC) phenotype post-treatment, reflecting a shift toward a more inflamed and potentially immunoresponsive tumor state [52].

### 3.6. Epigenetic Reactivation and Molecular Pathways

#### 3.6.1. Tumor Suppressor Reactivation

Decitabine (DAC) reactivates transcriptionally silenced tumor suppressor genes in TNBC through both direct DNA demethylation and indirect epigenetic remodeling (Table 6). Dahn et al., 2020 reported that low-dose DAC (0.5 mg/kg, i.p., administered in 3/5-day cycles) induced the expression of multiple tumor suppressors including RUNX3, CDH1, and BRCA1 in TNBC xenografts derived from MDA-MB-231, MDA-MB-468, and SUM159 cell lines [47]. Notably, this reactivation did not consistently correlate with promoter demethylation, indicating that DAC may act through intermediate transcriptional regulators or by altering chromatin accessibility. For example, BRCA1 expression was restored in MDA-MB-231 and SUM159 models, even when its promoter remained partially methylated. This suggests that DAC-induced suppression of DNA methyltransferases (DNMTs), rather than specific CpG demethylation, may relieve repressive chromatin structures and allow transcription factor recruitment [47].

Yu et al., 2018 provided further mechanistic insight by showing that DAC promotes post-transcriptional degradation of DNMT1, DNMT3A, and DNMT3B proteins in both PDX tumors and patient-derived organoids, without significantly reducing DNMT mRNA levels [45]. This effect was mediated by TRAF6, an E3 ubiquitin ligase that was itself transcriptionally upregulated following DAC exposure. TRAF6 facilitated lysosome-dependent DNMT degradation, leading to a broad relief of methylation-mediated gene silencing. Importantly, DAC-sensitive PDX models displayed robust DNMT protein depletion, while resistant tumors (MCD-05) retained high DNMT expression despite DAC treatment. Knockout of TRAF6 in otherwise sensitive models abrogated DNMT degradation and tumor suppressor reactivation, thereby confirming its role as a gatekeeper of DAC efficacy [45].

In the study by Vernier et al., 2020, DAC was used in combination with C29, an ERRα inhibitor, in MDA-MB-231 xenografts [71]. This dual treatment reactivated IRF4, a transcription factor frequently silenced in TNBC, by disrupting a positive feedback loop between DNMT1 and ERRα. Silencing IRF4 reversed the anti-tumor effects of the combination, demonstrating its central role in tumor suppression. These data suggest that DAC may unlock a broader network of tumor suppressor programs when paired with additional transcriptional modulators [71].

#### 3.6.2. Post-Transcriptional Regulation

In addition to DNA methylation, DAC also affects post-transcriptional gene regulation, especially via non-coding RNAs. Chu et al., 2023 demonstrated that DAC treatment restored expression of miR-708, a tumor-suppressive microRNA commonly silenced in TNBC due to promoter hypermethylation [60]. Re-expression of miR-708 led to the downregulation of several oncogenic targets including Rap1B, IKKβ, and CD44, all of which contribute to tumor proliferation, invasion, and stemness. These effects were observed both in vitro (MDA-MB-231 cells) and in vivo, where DAC-treated xenografts exhibited decreased tumor growth and lower expression of these oncogenes. Notably, luciferase reporter assays confirmed direct binding between miR-708 and the 3′UTRs of Rap1B and CD44 mRNAs, supporting a direct regulatory mechanism [60].

Yang et al., 2020 identified a second epigenetically regulated microRNA—miR-155—as a modulator of DAC resistance in TNBC stem-like cells (CD24^−^/CD44^+^ phenotype) [61]. miR-155 was significantly upregulated in DAC-resistant TNBC cells and patient tissues. It directly suppressed the expression of TSPAN5, a membrane protein involved in endosomal trafficking and CD44 degradation. Downregulation of TSPAN5 led to the accumulation of CD44, reinforcing the stem-like phenotype and conferring resistance to DAC-induced differentiation. Restoration of TSPAN5 expression or inhibition of miR-155 reversed this resistance phenotype, reduced sphere formation, and sensitized TNBC cells to DAC. These findings suggest that post-transcriptional control of resistance-associated genes, particularly through miRNA-mediated repression, plays a critical role in modulating DAC efficacy in TNBC [61].

### 3.7. Other Combination Strategies to Enhance Therapeutic Efficacy

#### 3.7.1. Hormonal Therapies

Though TNBC is typically defined by a lack of hormone receptor expression, epigenetic silencing of ERα and ERβ contributes to this phenotype. Salahuddin et al., 2022 tested whether DAC could re-sensitize MDA-MB-231 TNBC cells to hormonal signaling through epigenetic reactivation [74]. DAC (4 µM) alone restored mRNA expression of ERα and ERβ, while co-treatment with the HDAC inhibitor vorinostat (0.26 µM) further enhanced chromatin accessibility. The addition of the selective ERβ agonist DPN (0.093 µM) yielded the most potent effects. The triple therapy (DAC + vorinostat + DPN) resulted in a 56-fold increase in ERβ mRNA, suppression of VEGF, IGF-1, and Cyclin D1 (measured via ELISA), and increased apoptosis (Caspase-3 activity). These findings suggest that DAC can reprogram the epigenome to restore endocrine receptor expression and, in combination with receptor agonists, suppress angiogenesis and tumor proliferation [74].

#### 3.7.2. Chemotherapy

Several studies have highlighted the synergistic effects of decitabine in combination with chemotherapeutic agents in TNBC, enhancing efficacy and potentially reducing required chemotherapy doses. Yu et al., 2018 investigated the capacity of decitabine to overcome chemoresistance using a panel of 15 patient-derived xenograft (PDX)-derived organoid models [45]. In chemo-refractory tumors, low-dose decitabine (100 nM for 7 days) induced TRAF6-mediated lysosomal degradation of DNMT1, DNMT3A, and DNMT3B, leading to demethylation and reactivation of silenced genes. Pre-treatment with decitabine significantly sensitized paclitaxel-resistant organoids to subsequent paclitaxel exposure. Importantly, PDX tumors with high DNMT protein expression responded to combination therapy, whereas TRAF6-deficient models remained resistant, confirming the necessity of DNMT degradation for chemosensitization [45]. Complementary findings were observed by Xiong et al., 2022 who showed that decitabine combined with docetaxel reactivated the tumor suppressor DAB2IP, thereby modulating the AKT and β-catenin signaling axes and enhancing apoptotic responses [69]. Similarly, Gao et al., 2022 employed a triple combination of decitabine, paclitaxel, and anti-PD-1 antibody, which elicited robust immunogenic cell death, immune priming, and complete tumor rejection in 75% of treated mice [65]. Building on this, He et al., 2022 utilized a nanoparticle-based co-delivery system for decitabine and paclitaxel, administered alongside anti-PD-L1 [68]. This combination achieved maximal tumor suppression and prolonged survival, attributed to epithelial–mesenchymal transition (EMT) reversal and enhanced immune cell infiltration [68]. These findings indicate that DAC–chemotherapy combinations act through complementary mechanisms—including epigenetic reprogramming, tumor suppressor gene reactivation, signaling modulation, and immune activation—enhancing therapeutic efficacy while potentially allowing lower chemotherapy doses.

### 3.8. Mechanisms of Resistance to Decitabine

Resistance to decitabine in TNBC involves a combination of epigenetic, post-translational, pharmacologic, and non-coding RNA-mediated mechanisms, which collectively impair its ability to induce tumor suppressor reactivation and immune remodeling. A prominent resistance mechanism identified by Yu et al., 2018 is the failure to degrade DNMT1, DNMT3A, and DNMT3B proteins following DAC treatment [45]. In sensitive TNBC models, DAC induces TRAF6-mediated K63-linked ubiquitination of these DNMTs, targeting them for lysosomal degradation. However, resistant models (such as MCD-04 and MCD-05) exhibit insufficient upregulation of TRAF6, allowing DNMT protein persistence despite DAC exposure. This blocks epigenetic reprogramming, maintains promoter methylation, and prevents re-expression of key regulatory genes [45].

A second key resistance mechanism involves deficient activation of decitabine via reduced expression of deoxycytidine kinase (DCK), the enzyme responsible for converting DAC into its active triphosphate form. Dahn et al., 2020 showed that TNBC xenografts with low DCK expression exhibit poor responses to DAC due to inadequate intracellular drug activation [47]. Notably, DCK expression increases post-chemotherapy, suggesting that DAC may be more effective in chemo-refractory TNBC populations. Nevertheless, in tumors with constitutively low DCK, pharmacologic resistance remains a barrier to DAC efficacy [47].

Third, post-transcriptional mechanisms mediated by microRNAs also drive DAC resistance, particularly in cancer stem cell-enriched TNBC subpopulations. Yang et al., 2020 identified miR-155 as a critical regulator of DAC resistance in CD44^+^/CD24^−^ stem-like TNBC cells. miR-155 represses TSPAN5, a membrane tetraspanin involved in the degradation of CD44. Downregulation of TSPAN5 by miR-155 leads to CD44 stabilization and enhanced self-renewal capacity, promoting stemness and resistance to DAC-induced differentiation [61]. Restoration of TSPAN5 or inhibition of miR-155 re-sensitized resistant cells to DAC, suggesting that this axis could be exploited to overcome stemness-associated resistance [61].

Importantly, several non-contributory factors were also identified, helping to refine predictive biomarkers. Dahn et al., 2020 reports that resistance was not associated with ABCB1 overexpression (a transporter linked to taxane resistance), nor with global DNA methylation patterns, nor with suppression of viral mimicry genes such as RIG-I or MDA5. For example, knockdown of MDA5 or RIG-I had no effect on DAC sensitivity, indicating that ERV-driven immune signaling is not required for DAC response in vitro [47].

Finally, resistance may also stem from the absence of gene silencing at key pro-apoptotic loci, as described by Nakajima et al., 2022 [66]. In “R-type” TNBC cells (such as HCC1937), minimal methylation of genes like NOXA precludes DAC-mediated reactivation, rendering the cells intrinsically resistant to epigenetic therapy. In these contexts, DAC lacks therapeutic substrate and produces little cytotoxicity, highlighting a form of primary resistance due to lack of epigenetic suppression [66].

### 3.9. Safety and Tolerability

Across various in vivo and in vitro experimental models including murine 4T1 models, xenografts, and patient-derived xenografts (PDXs), low-dose decitabine consistently induced epigenetic reprogramming with minimal toxicity. In murine studies, including those by Russo et al., 2024 and Chu et al., 2023, no significant adverse effects were observed during decitabine monotherapy or when used in combination with immunomodulatory agents like PeptiCRAd or dexamethasone [59,60]. Mouse body weight remained stable, and no overt signs of systemic toxicity or organ dysfunction were reported, indicating good tolerability during prolonged dosing regimens [59,60].

In a human phase II clinical trial by Bear et al., 2025, decitabine administered in combination with pembrolizumab was also well tolerated in TNBC patients [52]. Meanwhile, some immune-related adverse events were reported such as hypothyroidism (6.5%), adrenal insufficiency (14.3% in Cohort A2), and rare cases of vasculitis and Guillain–Barré syndrome. The majority of patients completed treatment without dose-limiting toxicities. Importantly, decitabine-associated myelosuppression, a known risk in hematologic malignancies, was not prominently observed in these solid tumor studies, likely due to the lower and intermittent dosing strategies employed [52].

Overall, decitabine appears to be a safe and tolerable epigenetic agent in TNBC models, with minimal systemic toxicity at effective doses [68,69]. This favorable safety profile supports its continued investigation as a priming agent for immunotherapy or chemotherapy, particularly in combination regimens aimed at reversing tumor immune evasion and enhancing therapeutic efficacy.

### 3.10. Histological and Phenotypical Changes

Decitabine induces distinct histologic remodeling within TNBC tumors, both in preclinical models and clinical settings. In murine studies, such as Russo et al., 2024, combination therapy with decitabine and PeptiCRAd resulted in notable spatial reorganization of intratumoral CD8^+^ T cells, shifting from perivascular zones to deeper tumor regions, an effect associated with enhanced immune surveillance [59]. This was accompanied by a reduction in epithelial markers such as pan-cytokeratin and E-cadherin, suggesting tumor de-differentiation or phenotypic plasticity under epigenetic pressure [59]. Similarly, in the clinical trial by Bear et al., 2025, decitabine combined with pembrolizumab increased both stromal and intratumoral tumor-infiltrating lymphocytes (TILs) and elevated PD-L1 expression, reflecting a transition toward a more inflamed tumor microenvironment [52]. Though traditional histopathologic grading was limited in some studies, indirect evidence from tumor volume reduction and tissue architecture changes in patient-derived xenograft (PDX) models (Yu et al., 2018) further supports the histologic impact of decitabine in vivo [45].

On a phenotypic level, decitabine consistently reprograms TNBC cells toward increased immunogenicity and reduced malignancy. Wu et al., 2021 demonstrated that decitabine restores STING pathway activity in MYC-overexpressing tumors by inhibiting DNMT1-mediated promoter methylation, leading to upregulation of IFNβ, MHC-I, and chemokines that promote CD8^+^ T cell recruitment and granzyme B-mediated cytotoxicity [73]. Dahn et al., 2020 observed reactivation of tumor suppressor genes and viral mimicry pathways, such as OASL and ISG15, reflecting a broad epigenetic induction of anti-tumor stress and immune responses [47]. In vitro studies further highlight phenotypic shifts toward apoptosis and reduced angiogenesis; for instance, Salahuddin et al., 2022 reported increased caspase-3 activity and reduced VEGF and proliferation markers following decitabine-based combination treatment [74]. Additionally, decitabine re-expression of CDH1 (E-cadherin) and suppression of FOXM1, as seen in Kim et al., 2019, mark a phenotypic transition from mesenchymal and invasive states to more differentiated epithelial profiles [62]. Collectively, these findings underscore decitabine’s capacity to remodel both tumor architecture and cellular identity, enhancing susceptibility to immune-mediated clearance.

## 4. Discussion

### 4.1. Summary and Interpretation of Findings

This systematic review evaluated 25 studies exploring the role of decitabine (DAC) in triple-negative breast cancer (TNBC), integrating data from in vitro experiments, in vivo murine models, patient-derived xenografts (PDXs), and one early-phase clinical trials. Collectively, these studies reveal a consistent and multifaceted anti-tumor effect of DAC, mediated through both direct cytotoxicity and indirect modulation of the tumor immune and epigenetic landscape.

In vitro, DAC was shown to inhibit proliferation, reduce clonogenic potential, and induce apoptosis in a variety of TNBC cell lines. This was often accompanied by re-expression of silenced tumor suppressor genes such as *BRCA1*, *CDH1*, and *RUNX3*, as well as the activation of viral mimicry pathways involving *ISG15* and *OASL* [46]. These effects were sometimes potentiated through combination with histone deacetylase inhibitors (such as panobinostat or romidepsin) or hormonal modulators [62,63,74], suggesting a synergistic benefit of dual epigenetic targeting.

In murine models, DAC treatment significantly reduced tumor volume and delayed progression, with enhanced effects observed in combination with immune modulators such as PeptiCRAd or checkpoint inhibitors like anti-PD-1 [47,59,65,68]. In the study by Russo et al., 2024, DAC combined with PeptiCRAd not only suppressed tumor growth but also induced a spatial reorganization of intratumoral CD8^+^ T cells and reduced immune exhaustion phenotypes (PD-1^+^TIM-3^+^), signaling improved T cell functionality [59].

Translational studies in PDX models, such as those by Yu et al., 2018, further demonstrated the heterogeneity of DAC response across patient-derived tumors [45]. While sensitive models exhibited durable tumor suppression and DNMT degradation via TRAF6-mediated ubiquitination, resistant models maintained DNMT expression and showed rapid regrowth post-treatment. Importantly, DAC was able to sensitize some resistant models to paclitaxel, supporting its use as a chemotherapy primer [45].

Clinically, Bear et al., 2025 evaluated DAC as a neoadjuvant priming agent in combination with pembrolizumab in early-stage TNBC patients [52]. The combination led to statistically significant increases in stromal TILs and PD-L1 H-scores, alongside a reduction in systemic immunosuppression via decreased monocytic MDSCs. A pathologic complete response (pCR) rate of 40.7% was achieved, with minimal toxicity and promising short-term recurrence data. Histological analysis revealed conversion to lymphocyte-predominant phenotypes in some patients, echoing the immune inflamed profiles seen in preclinical models [52].

A deeper mechanistic understanding of how DAC modulates epigenetic programs in TNBC is essential for optimizing its therapeutic integration. DAC-induced hypomethylation not only reactivates silenced tumor suppressor genes but also intersects with critical signaling pathways that drive TNBC heterogeneity [62,66,69,71,73]. For example, demethylation of regulatory regions can modulate the PI3K/AKT, Wnt/β-catenin, and NF-κB axes, thereby influencing proliferation, apoptosis, and invasive behavior [81]. These context-dependent effects partly explain the heterogeneous responses observed across TNBC subtypes and underscore the importance of pathway-specific biomarker development.

### 4.2. Context Within Current Literature

These findings are consistent with the growing body of literature that positions DNA methyltransferase inhibitors (DNMTis) as immunomodulatory agents capable of reversing immune evasion and epigenetic silencing in solid tumors. Previous studies in non-small cell lung cancer [82], ovarian cancer [83], and colorectal cancer have shown that DAC and azacitidine can activate endogenous retroviral elements and type I interferon responses [84], thereby augmenting immunotherapy efficacy. The restoration of STING signaling in MYC-driven TNBC, as reported by Wu et al., 2021, adds mechanistic depth to this paradigm by linking oncogene-driven immune suppression to DNMT1-mediated epigenetic control [73].

Another emerging concept is the induction of viral mimicry by hypomethylating agents [85,86]. DAC-driven reactivation of normally silenced endogenous retroviral elements results in the accumulation of double-stranded RNA, which activates pattern recognition receptors such as RIG-I and MDA5 [87,88]. This triggers type I interferon signaling and upregulation of antigen presentation machinery, effectively converting the tumor into a “viral-like” state that is more visible to the immune system [87]. When combined with chemotherapy-induced immunogenic cell death or immune checkpoint blockade, this mechanism has the potential to amplify anti-tumor immunity in solid tumors, including TNBC.

Although the safety profile of DAC is relatively well established, placing it in a comparative framework provides added clinical perspective. Azacitidine, another hypomethylating agent, acts through similar epigenetic mechanisms but differs in pharmacokinetics, tolerability, and clinical performance, particularly in solid tumors, raising important considerations regarding agent selection [89]. In parallel, the advent of immune checkpoint inhibitors has set a new standard for immunomodulatory efficacy in TNBC. Combining DAC with PD-1/PD-L1 blockade is especially compelling, as it integrates epigenetic reprogramming with durable immune activation, offering a strategy that could potentially enhance response rates and overcome resistance [65,73].

Additionally, DAC’s ability to sensitize tumors to chemotherapy and hormonal agents aligns with earlier findings in hematologic malignancies and estrogen receptor–silenced breast cancers [90,91]. The reactivation of *ERβ* in TNBC by Salahuddin et al., 2022 and subsequent suppression of angiogenesis and proliferation echoes observations that DAC can reverse receptor loss in epigenetically silenced breast tumors [74]. Together, these data affirm DAC’s role as a multi-modal reprogramming agent with broad applicability.

### 4.3. Preclinical and Translational Insights on Resistance

The emergence of resistance remains a consistent theme across DAC-related research [92]. The inability to degrade DNMT proteins, low expression of DCK (required for DAC activation), and upregulation of miRNAs like miR-155 (which stabilize stemness-related CD44 expression) all highlight the complexity of epigenetic resistance [61,93,94].

Preclinical studies have identified multiple mechanisms, including epigenetic silencing of pro-apoptotic genes (*NOXA* unsilencing) [66], oncogenic signaling pathways (FOXM1 upregulation; AKT/β-catenin activation) [62,69], and immune escape (STING pathway repression; interferon signaling inhibition) [71,73]. Several studies demonstrate that combinatorial approaches (for instance, DAC + HDAC inhibitors, DAC + immune checkpoint blockade) can partially overcome these mechanisms by reactivating silenced tumor suppressors and enhancing immunogenicity [62,63,65]. Additionally, targeting the miR-155 pathway through antisense oligonucleotides or miRNA sponges, as well as employing next-generation DNMT inhibitors with improved potency, represent promising strategies to overcome miRNA-mediated resistance and restore DAC sensitivity.

Translational models reveal additional complexities. Resistance phenotypes often correlate with failure to degrade DNMT proteins due to alterations in ubiquitination pathways (such as TRAF6 knockout) or impaired DAC activation from low deoxycytidine kinase (DCK) expression [47]. Moreover, maintenance of cancer stem-like traits via miRNA-mediated stabilization of CD44 sustains tumor cell plasticity and drug evasion [61]. These resistance mechanisms underscore the translational challenges in achieving durable DAC responses.

Interestingly, parallels exist between resistance mechanisms observed in TNBC and those described in hematologic malignancies such as myelodysplastic syndromes and acute myeloid leukemia, where reduced DCK activity and aberrant epigenetic regulator splicing contribute to DNMTi resistance [95,96].

### 4.4. Clinical Implications and Future Research Directions

The translational and clinical findings summarized in this review support a compelling role for decitabine as a priming agent in TNBC, particularly in combination with immune checkpoint inhibitors. DAC’s favorable safety profile, marked by minimal myelosuppression and manageable immune-related adverse events, makes it an attractive candidate for inclusion in neoadjuvant or perioperative regimens [97]. Furthermore, its capacity to epigenetically reprogram tumors into immunologically responsive states provides a rationale for its use in patients with otherwise immune-cold tumors. Beyond immunotherapy, DAC’s ability to restore endocrine receptor expression and sensitize tumors to chemotherapy expands its potential role in combination regimens [74].

Given the heterogeneity of DAC response in TNBC, successful clinical translation will require the development and validation of predictive biomarkers to enable patient stratification [98]. This process may follow a stepwise pipeline beginning with a discovery phase, where candidate biomarkers such as DNMT1 levels, DCK expression, TRAF6 status, interferon response signatures, and CD44-associated stemness markers are identified using high-throughput approaches like methylation profiling, RNA sequencing, and proteomics in DAC-sensitive versus resistant models. These findings should then be cross-referenced with large-scale clinical datasets such as TCGA and METABRIC for relevance. The subsequent analytical validation phase involves standardizing quantification techniques, including immunohistochemistry for DCK, methylation-specific PCR for BRCA1 and CDH1, and flow cytometry for tumor-infiltrating lymphocyte (TIL) profiling, potentially through multiplexed panels that capture both epigenetic and immune phenotypes. In the clinical validation phase, these assays are applied to biospecimens from completed or ongoing DAC-based trials to correlate biomarker status with treatment outcomes such as pathological complete response (pCR) or TIL dynamics. Finally, in the prospective utility phase, validated biomarkers should be integrated into the design of future clinical trials such as enrichment or adaptive trial models to assess their real-world predictive value and feasibility in both academic and community oncology settings.

Several clinical trials have explored the role of DAC and related DNMT inhibitors in TNBC, though not all have reached completion. The DETECT trial (NCT03295552), which assessed decitabine in combination with carboplatin for metastatic TNBC, was unfortunately terminated due to slow enrollment, highlighting challenges in trial accrual for biomodulatory agents [50]. Similarly, NCT05673200, a trial evaluating ASTX727 (an oral formulation of decitabine with cedazuridine) in combination with paclitaxel and pembrolizumab, was suspended, underscoring the need for stronger preliminary efficacy signals and improved patient engagement strategies [99]. However, some studies have reached completion and provide important insights; for example, NCT04134884, a study testing ASTX727 with the PARP inhibitor talazoparib in triple-negative or hormone-resistant/HER2-negative metastatic breast cancer, has completed recruitment and may offer valuable data on the feasibility and tolerability of dual epigenetic and DNA-damage response targeting [100]. These findings, taken together, reinforce both the promise and the operational challenges of integrating DAC into clinical practice.

In parallel, mechanistic studies should further elucidate the downstream consequences of DAC-induced transcriptional reprogramming, particularly its effects on tumor plasticity, epithelial–mesenchymal transition (EMT), and immune evasion in stem-like tumor subpopulations. Rational combination strategies must also be refined through trials evaluating DAC with immunotherapies, chemotherapies, hormonal agents, and other epigenetic modulators. This includes optimizing dosing schedules (such as pulsed vs. continuous), treatment sequencing (such as priming vs. concurrent), and maintenance regimens. Finally, immune profiling and spatial transcriptomic techniques such as single-cell RNA sequencing and multiplex immunohistochemistry should be leveraged to dissect how DAC reshapes the tumor-immune microenvironment at both cellular and spatial resolutions. Future trials would benefit from embedding these approaches into adaptive frameworks to better match patients to effective regimens, minimize exposure to ineffective treatments, and enhance overall trial feasibility.

### 4.5. Limitations

This systematic review has several limitations. First, the dominance of preclinical data (24 out of 25 studies) limits the immediate generalizability of findings to human populations. Second, methodological heterogeneity across studies, in terms of DAC dosing, treatment duration, and outcome measurement, which limits direct comparison and precludes meta-analysis. Third, long-term efficacy and recurrence data were inconsistently reported, particularly in in vivo models. Additionally, many mechanistic insights are based on a small number of models or cell lines, raising concerns about generalizability across the diverse spectrum of TNBC subtypes. The inclusion of only English-language publications minimizes interpretive errors, it may introduce publication bias by excluding relevant non-English data.

Early-phase studies have demonstrated biologic activity, including evidence of demethylation and immune activation, yet results have been tempered by challenges in patient selection, optimal dosing schedules, and the narrow therapeutic window imposed by myelosuppression. Moreover, sequential or metronomic dosing strategies are being explored to balance efficacy with tolerability [101].

## 5. Conclusions

Decitabine exerts multifaceted anti-tumor effects in triple-negative breast cancer through epigenetic reprogramming, immune activation, and restoration of tumor suppressor function presented here as Graphical Abstract. Preclinical and early clinical data support its role as a safe and effective adjunct to immunotherapy, chemotherapy, and hormonal agents. Its favorable safety profile and ability to prime tumors into immunologically responsive states further highlight its translational potential in neoadjuvant or perioperative settings. However, resistance mechanisms and patient heterogeneity present ongoing challenges to its broad implementation.

To fully realize the therapeutic potential of DAC in TNBC, future research must prioritize biomarker-driven [73] clinical trials, mechanistic validation in diverse patient populations, and optimization of combination strategies. If these hurdles are addressed, decitabine could emerge as a valuable component of precision oncology approaches in this hard-to-treat breast cancer subtype.

## Figures and Tables

**Table 1:** PICOS framework.

Framework	Description
P (Participants)	Patients with triple-negative breast cancer (TNBC); preclinical in vivo and in vitro models involving TNBC cell lines or xenografts.
I (Intervention)	Administration of decitabine (5-aza-2′-deoxycytidine), either as monotherapy or in combination with other therapeutic agents.
C (Comparison)	Standard chemotherapeutic agents, placebo, or untreated control groups.
O (Outcomes)	Tumor progression metrics (overall response rate, progression-free survival, overall survival, disease-free survival), biomarker responses, immune response, epigenetic modulation, histopathological changes, safety profile.
S (Study Design)	Preclinical in vivo/in vitro studies and clinical studies (including randomized trials, observational studies, and case series) evaluating the role of decitabine in TNBC.

**Table 2:** In vitro evidence of cytotoxicity and clonogenic suppression.

Study	Cell Lines Used	Intervention	Key Findings
Dahn et al., 2020 [47]	MDA-MB-468, MDA-MB-231, SUM159	0.5 mg/kg DAC for 3/5-day cycles over 3–4 weeks	Dose-dependent cytotoxicity; significant reduction in clonogenic potential; apoptosis induction; observed dependence on DCK expression levels for drug activation.
Chu et al., 2023 [60]	MDA-MB-231, HCC-1395, Hs578T, MCF-10A (non-tumorigenic), MCF-7	50 mg/kg DAC 5 mg/kg DEX	Upregulation of miR-708; suppression of CD44 and Rap1B expression; inhibited migration, invasion, and clonogenicity.
Salahuddin et al., 2022 [74]	MDA-MB-231	4 µM DAC 0.26 µM Vorinostat 0.093 µM DPN (ERβ agonist) Combinations: Decitabine + Vorinostat, DPN + Vorinostat, DPN + Decitabine, and all three together	Hormonal resensitization through ERβ reactivation; increased apoptotic signaling via caspase-3 elevation; VEGF downregulation.
Yang et al., 2020 [61]	MDA-MB-231, BT-549	DAC (concentrations ranged from 2 μmol/L to 256 μmol/L) + Modulatory agents (miR-155 mimics, miR-155 inhibitors, siTSPAN5, pcDNA3.1-TSPAN5 plasmids)	DAC increased miR-155, promoting resistance via TSPAN5 suppression; co-treatment with miR-155 inhibitor restored TSPAN5 and reduced tumorsphere formation.
Kim et al., 2019 [62]	MDA-MB-231, MDA-MB-436, MDA-MB-468, Hs578T, HCC1806, HCC1569, DU4475	5 μM DAC + 120 nM PAN for 24–48 h	TNBC-specific cytotoxicity; CDH1 upregulation and FOXM1 suppression; induced cell cycle arrest at G2/M phase.
Cooper et al., 2012 [63]	MDA-MB-231, BT20	1 μM DAC for 72 h + 5 nM Romidepsin during the last 24 h	Reduced colony-forming ability; synergistic cytotoxicity with HDAC inhibitor; reactivation of Wnt antagonist sFRP1.
Vernier et al., 2020 [71]	MDA-MB-231, MDA-MB-436, MDA-MB-468	5 μM C29 + 3–5 μM AZA	Reduced proliferation; de-repression of IRF4 via disruption of DNMT1–ERα loop; induction of type I interferon-related genes.

**Table 3:** Tumor volume reduction in murine models.

Study	Murine Model	Intervention	Key Findings
Dahn et al., 2020 [47]	MDA-MB-468, MDA-MB-231, SUM159	0.5 mg/kg DAC for 3/5-day cycles over 3–4 weeks	Significant tumor volume reduction (~60%); effect reversed in DCK-deficient tumors.
Russo et al., 2024 [59]	4T1	0.5 mg/kg DAC + PeptiCRAd (1 × 10^9^ viral particles per tumor + 20 µg peptide)	Significant tumor shrinkage in DAC + PeptiCRAd group compared to monotherapies.
Wu et al., 2021 [73]	66cl4, 4T1	0.8 mg/kg DAC daily + 100 µ Anti-PD-1 antibody on days 3, 7, 10, 14	Combination significantly reduced tumor burden compared to monotherapy.
Gao et al., 2022 [65]	4T1	10 mg/kg PTX + 2.5 mg/kg DAC + 5 mg/kg aPD-1	DAC + PTX + aPD-1 resulted in tumor rejection in 75% of mice.
Banerjee et al., 2023 [76]	4T1	6.25 mg/kg 5-ADC + 15 mg/kg Verteporfin	Combo group showed smallest tumor volumes.
He et al., 2024 [68]	4T1 and PTX-resistant 4T1/PTX	30 µM DAC + 87.69 µM PTX + 0.3 mg/kg αPD-L1	Greatest tumor volume reduction in triple combo group.
Vernier et al., 2020 [71]	NIC-5231, NIC-5257	5 μM C29 + 3–5 μM 5-azadC	DAC + C29 group showed maximal tumor suppression.

**Table 4:** Antigen presentation and T-cell recruitment.

Study	Model	Intervention	Immune Markers Affected	Other Notes
Bear et al., 2020 [52]	Primary breast tumor tissue (biopsy-derived)	15 mg/m^2^ DAC × 4 days 200 mg Pembrolizumab on days 8 and 22	↑ sTILs, ↑ PD-L1 (H-score, CPS), ↓ M-MDSCs	Correlated with pCR; effective immune recruitment.
Russo et al., 2024 [59]	4T1, MDA-MB-436	0.5 mg/kg DAC + PeptiCRAd (1 × 10^9^ viral particles per tumor + 20 µg peptide)	↑ MHC-I, PD-L1, CD8^+^ T-cell infiltration; ↓ Tregs	Enhanced spatial relocalization of CD8^+^ T cells; tumor growth control.
Wu et al., 2021 [73]	66cl4, 4T1	0.8 mg/kg DAC daily + 100 µ Anti-PD-1 antibody on days 3, 7, 10, 14	↑ IFN-β, ISGs (CXCL10); ↑ CD8^+^ T cells	Strong STING pathway activation; immune inflamed phenotype.
Gao et al., 2022 [65]	4T1	10 mg/kg PTX + 2.5 mg/kg DAC + 5 mg/kg aPD-1	↑ IFN-γ, CD8^+^, Granzyme B+ cells	Induced ICD and tumor clearance in 75% of mice.

**Table 5:** Combination with immune checkpoint inhibitors.

Study	Model	Intervention	Synergistic Effects	Other Notes
Bear et al., 2020 [52]	Primary breast tumor tissue (biopsy-derived)	15 mg/m^2^ DAC × 4 days 200 mg Pembrolizumab on days 8 and 22	40.7% pCR rate, ↑ sTILs, ↓ M-MDSCs	Correlated with pCR; effective immune recruitment.
Wu et al., 2021 [73]	66cl4, 4T1	0.8 mg/kg DAC daily + 100 µ Anti-PD-1 antibody on days 3, 7, 10, 14	Enhanced IFN-I and CD8^+^ response; greater tumor volume reduction vs. monotherapy.	MYC-DNMT1-STING axis central to synergy.
Gao et al., 2022 [65]	4T1	10 mg/kg PTX + 2.5 mg/kg DAC + 5 mg/kg aPD-1	Tumor rejection in 75% of mice.	Synergized with chemo and immune priming effect.
He et al., 2024 [68]	4T1 and PTX-resistant 4T1/PTX	30 µM DAC + 87.69 µM PTX + 0.3 mg/kg αPD-L1 (in imaging studies)	Triple combo showed best survival and tumor suppression.	EMT reversal enhanced checkpoint efficacy.

**Table 6:** Tumor suppressor reactivation.

Study	Model	Tumor Suppressor Activated	Mechanisms	Other Notes
Yu et al., 2019 [46]	Hs 578T, BT-549, MDA-MB-231	Global gene reprogramming including MYC downregulation	TRAF6-mediated DNMT degradation.	Broad reactivation of suppressed pathways; resensitization to chemotherapy.
Dahn et al., 2020 [47]	MDA-MB-468, MDA-MB-231, SUM159	BRCA1, CDH1, RUNX3	Partial promoter demethylation; viral mimicry.	Linked to DAC activation via DCK; reduced tumor volume.
Cooper et al., 2012 [63]	MDA-MB-231, BT20	sFRP1	Histone and DNA demethylation with DAC + Romidepsin.	Wnt pathway inhibition; induced apoptosis.
Fan et al., 2024 [64]	BT-549, HCC1937, MDA-MB-231	EMT-related markers (such as E-cadherin)	Dual HDAC and DNMT inhibition.	Reduced migration and invasiveness.
Nakajima et al., 2022 [66]	D-type: MDA-MB-468, HCC38 G-type: MDA-MB-453, MDA-MB-157, MDA-MB-231, HCC1143 R-type: Hs578T, HCC1187, HCC1937	NOXA (pro-apoptotic gene)	Epigenetic class profiling; unsilenced in R-type.	Correlated with resistance; failure to silence pro-apoptotic gene in resistant class.
Vernier et al., 2020 [71]	MDA-MB-231, MDA-MB-436, MDA-MB-468	IRF4	Disruption of ERα-DNMT1 complex.	Enhanced interferon signaling and growth suppression.

## Data Availability

The datasets analyzed during this study are publicly available in PubMed, EBSCO, Web of Science, and Semantic Scholar up to 26 April 2025. The search strategy included the following keywords and Boolean operators: (“Decitabine” OR “Azacitidine” OR “DNMT inhibitor” OR “5-aza-2′-deoxycytidine” OR “DNA hypomethylating agents”) AND (“TNBC” OR “Triple-negative breast cancer”). The full search was conducted manually and is detailed in the Section 2. No additional ethical approval was required as the study utilized publicly available data.

## Supplementary Materials

The following supporting information can be downloaded at https://www.mdpi.com/article/10.3390/cancers17182953/s1; Table S1: PRISMA-2020 checklist; Table S2: AMSTAR-2 checklist; Table S3: Risk of bias assessment using the Quality in Prognosis Studies (QUIPS) tool; Table S4: Risk of bias assessment using the Cochrane RoB-2 tool; Table S5: Study characteristics.

## Abbreviations

The following abbreviations are used in this manuscript:

TNBC	Triple-Negative Breast Cancer
AKT	Protein Kinase B
BRCA	Breast Cancer Genes
CDH-1	E-Cadherin
CREB1	cAMP Response Element Binding Protein 1
DAB2IP	Disabled Homolog 2 Interacting Protein
DNMT/DNMT1	DNA Methyltransferase
DNMTi	DNA Methyltransferase Inhibitor
DPN	Diarylpropionitrile
ERα	Estrogen Receptor Alpha
ERβ	Estrogen Receptor Beta
ERBB2	Erb-B2 Receptor Tyrosine Kinase 2
ESSRA	Estrogen-Related Receptor Alpha
FOXM1	Forkhead Box Protein M1
FTH1	Ferritin Heavy Chain 1
GSK	Glycogen Synthase Kinase-3
HMGB1	High-Mobility Group Box 1
HRR	Homologous Recombination Repair
hTERT	Human Telomerase Reverse Transcriptase
IGF-1	Insulin-like Growth Factor 1
IKKb	Inhibitor of Nuclear Factor Kappa-B Kinase Subunit Beta
IRF4	Interferon Regulatory Factor 4
LPAR1	Lysophosphatidic Acid Receptor 1
LRRC26	Leucine-Rich Repeat-Containing Protein 26
MYC	MYC Proto-Oncogene, bHLH Transcription Factor
NOXA	Phorbol-12-myristate-13-acetate-Induced Protein 1
NUPR1	Nuclear Protein 1
PARP	Poly (ADP-ribose) Polymerase
PDT	Photodynamic Therapy
PIK3CB	Phosphoinositide 3-Kinase Catalytic Subunit Beta
POLD3	Polymerase Delta 3
Rap1B	Ras-Related Protein 1b
RUX3	Runt-Related Transcription Factor 3
sFRP1	Secreted Frizzled-Related Protein 1
STING	Stimulator of Interferon Genes
TP53	Tumor Protein P53
TPX2	Targeting Protein for Xklp2
TRAF6	Tumor Necrosis Factor Receptor-Associated Factor 6
TSPAN5	Tetraspanin 5
VEGF	Vascular Endothelial Growth Factor
AZA	Azacitidine
DAC	Decitabine
ADC	5-aza-2′-deoxycytidine
DEX	Dexamethasone
DOC	Docetaxel
PAN	Panobinostat
PTX	Paclitaxel
ATP	Adenosine Triphosphate
CRT	Chemoradiotherapy
CSCs	Cancer Stem Cells
DNA	Deoxyribonucleic Acid
DSBs	Double Strand Breaks
EMT	Epithelial–Mesenchymal Transition
EPR	Enhanced Permeability and Retention
γ-H2AX	Phosphorylated Form of Histone H2AX
miR	microRNA
lncRNA	Long Non-Coding RNA
shRNA	Short Hairpin RNA
CTLs	Cytotoxic T Lymphocytes
ICD	Immunogenic Cell Death
IFN-γ	Interferon Gamma
IFN-β	Interferon Beta
IFN-I	Interferon Type I
ISGs	Interferon-Stimulated Genes
MHC-I	Major Histocompatibility Complex Class I
M-MDSCs	Monocytic Myeloid-Derived Suppressor Cells
PD-1	Programmed Cell Death Protein 1
PD-L1	Programmed Cell Death Protein Ligand 1
Treg	Regulatory T Cells
TEM	Effector Memory T Cells
ALT	Alternative Lengthening of Telomeres
DFS	Disease-Free Survival
LNEs	Lipid Nanoemulsions
mMSNs	Macrophage-Membrane-Camouflaged Mesoporous Silica Nanoparticles
NCT	Neoadjuvant Chemotherapy
OS	Overall Survival
pCR	Pathological Complete Response
sTILs	Stromal Tumor-Infiltrating Lymphocytes
iTILs	Intratumoral Tumor-Infiltrating Lymphocytes
CRISPR	Clustered Regularly Interspaced Short Palindromic Repeats
DMEM	Dulbecco’s Modified Eagle Medium
