# Supplementary material for: Epigenetic Reprogramming by Decitabine in Triple-Negative Breast Cancer: Mechanisms, Immune Modulation, and Therapeutic Synergy
Source: Cancers (Basel). 2025 Sep 9;17(18):2953. doi: 10.3390/cancers17182953 (PMC12468927; doi:10.3390/cancers17182953)
Supplement: Supplementary file 1 [file cancers-17-02953-s001.zip › cancers-3824457-supplementary.pdf]

Table S1. PRISMA-2020 checklist.

Section and Topic	Item #	Checklist item	Location where item is reported
**TITLE**			
Title	1	Identify the report as a systematic review.	Lines 2-4
**ABSTRACT**			
Abstract	2	See the PRISMA 2020 for Abstracts checklist.	Lines 27-47
**INTRODUCTION**			
Rationale	3	Describe the rationale for the review in the context of existing knowledge.	Lines 54-114
Objectives	4	Provide an explicit statement of the objective(s) or question(s) the review addresses.	Lines 115-124
**METHODS**			
Eligibility criteria	5	Specify the inclusion and exclusion criteria for the review and how studies were grouped for the syntheses.	Lines 148-182
Information sources	6	Specify all databases, registers, websites, organisations, reference lists and other sources searched or consulted to identify studies. Specify the date when each source was last searched or consulted.	Lines 136-146
Search strategy	7	Present the full search strategies for all databases, registers and websites, including any filters and limits used.	Lines 136-146
Selection process	8	Specify the methods used to decide whether a study met the inclusion criteria of the review, including how many reviewers screened each record and each report retrieved, whether they worked independently, and if applicable, details of automation tools used in the process.	Lines 184-197

Data collection process	9	Specify the methods used to collect data from reports, including how many reviewers collected data from each report, whether they worked independently, any processes for obtaining or confirming data from study investigators, and if applicable, details of automation tools used in the process.	Lines 199-208
Data items	10a	List and define all outcomes for which data were sought. Specify whether all results that were compatible with each outcome domain in each study were sought (e.g. for all measures, time points, analyses), and if not, the methods used to decide which results to collect.	Lines 199-208
	10b	List and define all other variables for which data were sought (e.g. participant and intervention characteristics, funding sources). Describe any assumptions made about any missing or unclear information.	Lines 199-208
Study risk of bias assessment	11	Specify the methods used to assess risk of bias in the included studies, including details of the tool(s) used, how many reviewers assessed each study and whether they worked independently, and if applicable, details of automation tools used in the process.	Lines 210-245
Effect measures	12	Specify for each outcome the effect measure(s) (e.g. risk ratio, mean difference) used in the synthesis or presentation of results.	Lines 199-208
Synthesis methods	13a	Describe the processes used to decide which studies were eligible for each synthesis (e.g. tabulating the study intervention characteristics and comparing against the planned groups for each synthesis (item #5)).	Lines 148-182
	13b	Describe any methods required to prepare the data for presentation or synthesis, such as handling of missing summary statistics, or data conversions.	Lines 199-208
	13c	Describe any methods used to tabulate or visually display results of individual studies and syntheses.	Lines 199-208
	13d	Describe any methods used to synthesize results and provide a rationale for the choice(s). If meta-analysis was performed, describe the model(s), method(s) to identify the presence and extent of statistical heterogeneity, and software package(s) used.	No meta-analysis conducted
	13e	Describe any methods used to explore possible causes of heterogeneity among study results (e.g. subgroup analysis, meta-regression).	No meta-analysis conducted

	13f	Describe any sensitivity analyses conducted to assess robustness of the synthesized results.	No meta-analysis conducted
Reporting bias assessment	14	Describe any methods used to assess risk of bias due to missing results in a synthesis (arising from reporting biases).	Lines 210-245
Certainty assessment	15	Describe any methods used to assess certainty (or confidence) in the body of evidence for an outcome.	Lines 210-245
**RESULTS**			
Study selection	16a	Describe the results of the search and selection process, from the number of records identified in the search to the number of studies included in the review, ideally using a flow diagram.	Lines 248-272 + Figure 1
	16b	Cite studies that might appear to meet the inclusion criteria, but which were excluded, and explain why they were excluded.	Lines 248-272 + Figure 1
Study characteristics	17	Cite each included study and present its characteristics.	Lines 324-340 + Supplementary Material-3

Risk of bias in studies	18	Present assessments of risk of bias for each included study.	Lines 278-320 + Figures 2,3, and 4 + Supplementary Material-2
Results of individual studies	19	For all outcomes, present, for each study: (a) summary statistics for each group (where appropriate) and (b) an effect estimate and its precision (e.g. confidence/credible interval), ideally using structured tables or plots.	Supplementary Material-3
Results of syntheses	20a	For each synthesis, briefly summarise the characteristics and risk of bias among contributing studies.	Lines 278-320 + Figures 2,3, and 4 + Supplementary Material-2
	20b	Present results of all statistical syntheses conducted. If meta-analysis was done, present for each the summary estimate and its precision (e.g. confidence/credible interval) and measures of statistical heterogeneity. If comparing groups, describe the direction of the effect.	No meta-analyses conducted
	20c	Present results of all investigations of possible causes of heterogeneity among study results.	No meta-analyses conducted
	20d	Present results of all sensitivity analyses conducted to assess the robustness of the synthesized results.	No meta-analyses conducted
Reporting biases	21	Present assessments of risk of bias due to missing results (arising from reporting biases) for each synthesis assessed.	Lines 278-320 + Figures 2,3, and 4 + Supplementary Material-2
Certainty of evidence	22	Present assessments of certainty (or confidence) in the body of evidence for each outcome assessed.	Lines 278-320 + Figures 2,3, and 4 + Supplementary Material-2
**DISCUSSION**			

Discussion	23a	Provide a general interpretation of the results in the context of other evidence.	Lines 696-711
	23b	Discuss any limitations of the evidence included in the review.	Lines 793-803
	23c	Discuss any limitations of the review processes used.	Lines 793-803
	23d	Discuss implications of the results for practice, policy, and future research.	Lines 737-791
**OTHER INFORMATION**			
Registration and protocol	24a	Provide registration information for the review, including register name and registration number, or state that the review was not registered.	Not registered
	24b	Indicate where the review protocol can be accessed, or state that a protocol was not prepared.	Protocol described in detail in the Methodology section (pages 4-7)
	24c	Describe and explain any amendments to information provided at registration or in the protocol.	No amendments made
Support	25	Describe sources of financial or non-financial support for the review, and the role of the funders or sponsors in the review.	Lines 839-840
Competing interests	26	Declare any competing interests of review authors.	Line 854
Availability of data, code and other materials	27	Report which of the following are publicly available and where they can be found: template data collection forms; data extracted from included studies; data used for all analyses; analytic code; any other materials used in the review.	Lines 844-849

Page MJ, McKenzie JE, Bossuyt PM, Boutron I, Hoffmann TC, Mulrow CD, et al. The PRISMA 2020 statement: an updated guideline for reporting systematic reviews. BMJ 2021;372:n71. doi: 10.1136/bmj.n71. This work is licensed under CC BY 4.0. To view a copy of this license, visit https://creativecommons.org/licenses/by/4.0/

Table S2. AMSTAR-2 checklist.

1. **Did the research questions and inclusion criteria for the review include the components of PICO?**

For Yes:<br>☑ Population<br>☑ Intervention<br>☑ Comparator group<br>☑ Outcome	Optional (recommended):<br>☐ Timeframe for follow-up	☑ Yes<br>☐ No

2. **Did the report of the review contain an explicit statement that the review methods were established prior to the conduct of the review and did the report justify any significant deviations from the protocol?**

For Partial Yes:<br>The authors state that they had a written protocol or guide that included ALL the following:<br>☑ review question(s)<br>☑ a search strategy<br>☑ inclusion/exclusion criteria<br>☑ a risk of bias assessment	For Yes:<br>As for partial yes, plus the protocol should be registered and should also have specified:<br>☐ a meta-analysis/synthesis plan, if appropriate, and<br>☐ a plan for investigating causes of heterogeneity<br>☐ justification for any deviations from the protocol	☐ Yes<br>☑ Partial Yes<br>☐ No

3. **Did the review authors explain their selection of the study designs for inclusion in the review?**

For Yes, the review should satisfy ONE of the following:<br>☐ *Explanation for* including only RCTs<br>☑ OR *Explanation for* including only NRSI<br>☐ OR *Explanation for* including both RCTs and NRSI	☑ Yes<br>☐ No

4. **Did the review authors use a comprehensive literature search strategy?**

For Partial Yes (all the following):<br>The authors state that they had a written protocol or guide that included ALL the following:<br>☑ Searched at least 2 databases (relevant to research question)<br>☑ provided key word and/or search strategy<br>☑ justified publication restrictions (e.g.	For Yes, should also have (all the following):<br>☐ searched the reference lists/ bibliographies of included studies<br>☑ searched trial/ study registries<br>☐ included/consulted content experts in the field<br>☐ where relevant, searched for grey	☐ Yes<br>☑ Partial Yes<br>☐ No

language)	literature<br>☑ conducted search within 24 months of completion of the review	

5. **Did the review authors perform study selection in duplicate?**

For Yes, either ONE of the following:<br>☑ at least two reviewers independently agreed on selection of eligible studies and achieved consensus on which studies to include<br>☐ OR two reviewers selected a sample of eligible studies <u>and</u> achieved good agreement (at least 80 percent), with the remainder selected by one reviewer.	☑ Yes<br>☐ No

6. **Did the review authors perform data extraction in duplicate?**

For Yes, either ONE of the following:<br>☑ at least two reviewers achieved consensus on which data to extract from included studies<br>☐ OR two reviewers extracted data from a sample of eligible studies <u>and</u> achieved good agreement (at least 80 percent), with the remainder extracted by one reviewer.	☑ Yes<br>☐ No

7. **Did the review authors provide a list of excluded studies and justify the exclusions?**

For Partial Yes:<br>☐ provided a list of all potentially relevant studies that were read in full-text form but excluded from the full review	For Yes, must also have:<br>☐ justified the exclusion from the review of each potentially relevant study	☐ Yes<br>☐ Partial Yes<br>☑ No

8. **Did the review authors describe the included studies in adequate detail?**

For Partial Yes (ALL the following):<br>☑ described populations<br>☑ described interventions<br>☑ described comparators<br>☑ described outcomes<br>☑ described research	For Yes, should also have ALL the following:<br>☑ described population in detail<br>☑ described intervention in detail (including doses where relevant)<br>☑ described comparator in	☑ Yes<br>☐ Partial Yes<br>☐ No

designs	detail (including doses where relevant)<br>☑ described study's setting<br>☑ time frame for follow-up	

9. Did the review authors use a satisfactory technique for assessing the risk of bias (RoB) in individual studies that were included in the review?

**RCTs**		
For Partial Yes, must have assessed RoB from:<br>☐ unconcealed allocation, *and*<br>☐ lack of blinding of patients and assessors when assessing outcomes (unnecessary for objective outcomes such as all cause mortality)	For Yes, must also have assessed RoB from:<br>☐ allocation sequence that was not truly random, *and*<br>☐ selection of the reported result from among multiple measurements or analyses of a specified outcome	☐ Yes<br>☐ Partial Yes<br>☐ No<br>☑ Includes only NRSI
**NRSI**		
For Partial Yes, must have assessed RoB from:<br>☑ from confounding, *and*<br>☑ from selection bias	For Yes, must also have assessed RoB from:<br>☑ allocation sequence that was not truly random, *and*<br>☑ selection of the reported result from among multiple measurements or analyses of a specified outcome	☑ Yes<br>☐ Partial Yes<br>☐ No<br>☐ Includes only NRSI

10. Did the review authors report on the sources of funding for the studies included in the review?

For Yes,<br>☐ must have reported on the sources of funding for individual studies included in the review. Note: Reporting that the reviewers looked for this information but it was not reported by study authors also qualifies.	☐ Yes<br>☑ No

11. If meta-analysis was performed did the review authors use appropriate methods for statistical combination of results?

**RCTs:**<br>For Yes,<br>☐ the authors justified combining the data in a meta-analysis<br>☐ AND they used an appropriate weighted technique to combine study results and adjusted for heterogeneity if present<br>☐ AND investigated the causes of any heterogeneity	☐ Yes<br>☐ No<br>☑ No meta-analysis conducted
**NRSI:**<br>For Yes,<br>☐ the authors justified combining the data in a meta-analysis<br>☐ AND they used an appropriate weighted technique to combine study results and adjusted for heterogeneity if present<br>☐ AND they statistically combined effect estimates from NRSI that were adjusted for confounding, rather than combining raw data, or justified combining raw data when adjusted effect estimates were not available<br>☐ AND they reported separate summary estimates for RCTs and NRSI separately when both were included in the review	☐ Yes<br>☐ No<br>☑ No meta-analysis conducted

12. If meta-analysis was performed, did the review authors assess the potential impact of RoB in individual studies on the results of the meta-analysis or other evidence synthesis?

For Yes,<br>☐ included only low risk of bias RCTs<br>☐ OR, if the pooled estimate was based on RCTs and/or NRSI at variable RoB, the authors performed analyses to investigate possible impact of RoB on summary estimates of effect.	☐ Yes<br>☐ No<br>☑ No meta-analysis conducted

13. Did the review authors account for RoB in individual studies when interpreting/ discussing the results of the review?

For Yes,<br>☐ included only low risk of bias RCTs<br>☑ OR, if RCTs with moderate or high RoB, or NRSI were included the review provided a discussion of the likely impact of RoB on the results.	☑ Yes<br>☐ No

14. Did the review authors provide a satisfactory explanation for, and discussion of, any heterogeneity observed in the results of the review?

For Yes,	☑ Yes

☐ there was no significant heterogeneity in the results<br>☑ OR, if if heterogeneity was present the authors performed an investigation of sources of any heterogeneity in the results and discussed the impact of this on the results of the review	☐ No

15. If they performed quantitative synthesis, did the review authors carry out an adequate investigation of publication bias (small study bias) and discuss its likely impact on the results of the review?

For Yes,<br>☐ performed graphical or statistical tests for publication bias and discussed the likelihood and magnitude of impact of publication bias	☐ Yes<br>☐ No<br>☑ No meta-analyses conducted

16. Did the review authors report any potential sources of conflict of interest, including any funding they received for conducting the review?

For Yes,<br>☑ the authors reported no competing interests OR<br>☐ the authors described their funding sources and how they managed potential conflicts of interest	☑ Yes<br>☐ No

Shea BJ, Reeves BC, Wells G, Thuku M, Hamel C, Moran J, Moher D, Tugwell P, Welch V, Kristjansson E, Henry DA. AMSTAR 2: a critical appraisal tool for systematic reviews that include randomised or non-randomised studies of healthcare interventions, or both. BMJ. 2017 Sep 21;358:j4008.

Table S3. Risk of bias assessment using the Quality in Prognosis Studies (QUIPS) tool.

Study	Items and Score						Overall Risk
	1	2	3	4	5	6	
Yu et al., 2018 [45]	***	***	***	***	***	***	Low risk
Dahn et al., 2020 [47]	***	***	***	***	**	***	Low risk
Russo et al., 2024 [59]	***	***	***	***	**	***	Low risk
Chu et al., 2023 [60]	***	***	***	***	***	***	Low risk
Wu et al., 2021 [73]	***	***	***	**	**	***	Moderate risk
Salahuddin et al., 2022 [74]	***	***	***	**	**	***	Moderate risk
Yang et al., 2020 [61]	***	***	***	***	***	***	Low risk
Kim et al., 2019 [62]	***	***	***	***	**	***	Low risk
Cooper et al., 2012 [63]	***	***	***	***	***	***	Low risk
Fan et al., 2024 [64]	***	***	***	***	***	***	Low risk
Gao et al., 2022 [65]	***	***	**	***	**	***	Moderate risk
Butler et al., 2020 [75]	***	***	***	**	**	***	Moderate risk
Banerjee et al., 2023 [76]	***	***	**	**	**	***	Moderate risk
Nakajima et al., 2022 [66]	***	***	***	***	***	***	Low risk
Kong et al., 2015 [67]	***	***	***	**	***	***	Low risk
Miyagawa et al., 2018 [77]	***	***	***	***	**	**	Moderate risk
Wang et al., 2020 [78]	***	***	***	**	**	***	Moderate risk
Umeh-Garcia et al., [79]	***	***	***	***	***	***	Low risk

Study	Items and Score						Overall Risk
	**1**	**2**	**3**	**4**	**5**	**6**	
Yu et al., 2018 [45]	***	***	***	***	***	***	Low risk
Dahn et al., 2020 [47]	***	***	***	***	**	***	Low risk
Russo et al., 2024 [59]	***	***	***	***	**	***	Low risk
Chu et al., 2023 [60]	***	***	***	***	***	***	Low risk
Wu et al., 2021 [73]	***	***	***	**	**	***	Moderate risk
Al-dulaimi et al., 2024 [80]	***	***	**	**	*	**	High risk
He et al., 2024 [68]	***	***	***	***	**	***	Low risk
Xiong et al., 2022 [69]	***	***	***	***	***	***	Low risk
Elango et al., 2021 [70]	***	***	***	***	**	***	Low risk
Vernier et al., 2020 [71]	***	***	***	***	***	***	Low risk
Pacaud et al., 2025 [72]	***	***	***	***	***	***	Low risk

1. Study participation
2. Study attrition
3. Prognostic factor measurement
4. Outcome measurement
5. Study confounding
6. Statistical analysis and reporting

Table S4. Risk of bias assessment using the Cochrane RoB-2 tool.

Study	Items and Score					Overall Risk
	**1**	**2**	**3**	**4**	**5**	
Bear et al., 2025 [52]	***	***	***	***	***	Low risk

1. Bias arising from randomization process
2. Bias due to deviation from intended interventions
3. Bias due to missing outcome data
4. Bias in measurement of the outcome
5. Bias in selection of the reported result

Table S5. Study Characteristics.

Study	Country	Study Design	TNBC Cell Line	Intervention	Comparator	Key Findings
Yu et al., 2018 [45]	USA + China	Preclinical experimental study using patient-derived xenograft (PDX) models, organoids, and breast cancer cell lines (in vitro and in vivo)	Hs 578T, BT-549, MDA-MB-231	DAC	Vehicle	DNMT protein levels (not mRNA) strongly correlate with decitabine sensitivity.<br><br>DAC induces lysosome-dependent degradation of DNMTs via TRAF6.<br><br>High DNMT-expressing TNBCs are more responsive to decitabine.<br><br>DAC may sensitize tumors to chemotherapy (such as PTX)..<br><br>DNMT protein expression could serve as a predictive biomarker for treatment selection.
Dahn et al., 2020 [47]	Canada	Preclinical in vitro and in vivo experimental study using breast cancer cell lines and xenograft models	MDA-MB-468<br><br>MDA-MB-231<br><br>SUM159	DAC	Vehicle	DAC's efficacy is not predicted by methylation status or subtype.<br><br>DCK is critical for response; higher in TNBC and post-chemotherapy.<br><br>ABCB1 expression (common

						resistance mechanism to taxanes) does not reduce DACsensitivity.<br><br>Viral mimicry pathway activation occurred, but knockdown of MDA5/RIG-I did not alter sensitivity.<br><br>Suggests DAC is effective even in drug-resistant TNBC.
Bear et al., 2025 [52]	USA	Non-randomized, open-label, multi-center phase 2 trial	Primary breast tumor tissue (biopsy-derived)	DAC + Pembrolizumab (anti-PD-1 checkpoint inhibitor)	No control	Pre-NCT immune priming with DAC + pembrolizumab increased sTIL, iTIL, PD-L1 expression.<br><br>pCR achieved in 40.7% of TNBC patients..<br><br>Window treatment reduced M-MDSCs significantly.<br><br>Combination was well-tolerated after dose adjustment.
Russo et al., 2024 [59]	Finland	Preclinical in vivo and in vitro experimental study	4T1 (murine TNBC)<br><br>MDA-MB-436 (human	Low-dose DAC (epigenetic agent)	Vehicle	Combination therapy improved tumor control in 4T1 TNBC model.

			TNBC)	PeptiCRAd (peptide-coated adenoviral cancer vaccine)		DAC induced MHC-I and PD-L1 expression.<br><br>PeptiCRAd reduced Tregs and exhaustion markers.<br><br>CD8+ T cells showed spatial reorganization and improved cytotoxicity in 4T1.<br><br>Antitumor response was tumor-specific and linked to immunogenicity.
Chu et al., 2023 [60]	Taiwan	Preclinical in vivo and in vitro experimental study using xenograft mouse model and cell lines	MDA-MB-231<br><br>HCC-1395<br><br>Hs578T<br><br>MCF-10A (non-tumorigenic)<br><br>MCF-7	DAC<br><br>DEX<br><br>DAC + DEX	Vehicle	Combination of DAC and DEX significantly suppressed tumor growth and metastasis in TNBC xenograft model.<br><br>The therapy reactivated tumor suppressor pathways and miR-708 expression.<br><br>Protein levels of key oncogenic markers (Rap1B, CD44, IKKb) were reduced.

						The regimen is composed of FDA-approved drugs, potentially accelerating clinical translation.
Wu et al., 2021 [73]	China	Preclinical experimental study including in vitro cell culture, in vivo mouse models, and retrospective analysis of patient tumor samples	MDA-MB-231, BT-549, Hs578T, 66cl4, 4T1, HEK-293T, HCC1143	MYC knockdown (shRNA)<br><br>MYC overexpression (plasmid)<br><br>Anti–PD-1 antibody therapy	Vehicle or isotype control antibodies	MYC amplification suppresses innate immunity in TNBC via DNMT1-mediated silencing of STING.<br><br>Decitabine reverses immune suppression and enhances PD-1 blockade efficacy.<br><br>Proposed a novel combination immunotherapy approach for MYC-high TNBC tumors.
Salahuddin et al., 2022 [74]	Egypt	In vitro experimental study (non-randomized) using human TNBC cell line (MDA-MB-231)	MDA-MB-231 (ERα-/ERβ-, triple-negative breast cancer model)	DAC<br><br>Vorinostat (HDAC inhibitor)<br><br>DPN<br><br>Combinations: DAC + Vorinostat, DPN +	DMEM control medium	ERα and ERβ re-expression successfully achieved with DAC + Vorinostat.<br><br>ERβ activation via DPN enhanced anti-proliferative and pro-apoptotic effects.

				Vorinostat, DPN + DAC, and all three together		Triple therapy showed:<br>Highest ERβ expression (56-fold ↑)<br>Strongest suppression of Cyclin D1 and IGF-1<br>Highest Caspase-3 activity<br>Greatest VEGF suppression<br><br>Triple combination therapy holds promise as an epigenetic-hormonal approach for ER-negative TNBC.
Yang et al., 2020 [61]	China	In vitro experimental study	MDA-MB-231, BT-549	Primary: DAC<br><br>Modulatory agents: miR-155 mimics, miR-155 inhibitors, siTSPAN5, pcDNA3.1-TSPAN5 plasmids	Vehicle control, miR-NC (negative control), empty vector (pcDNA3.1), or untreated	miR-155 is upregulated in TNBC tissues, cell lines, and stem cell populations ($CD24^{-}/CD44^{+}$).<br><br>Overexpression of miR-155:<br>Increases stemness (SOX2, NANOG, mammosphere formation).<br>Increases resistance to DAC.<br>TSPAN5 is a direct target of miR-155, and its overexpression reverses these effects.<br>Knockdown of TSPAN5 abrogates the benefit of miR-155 inhibition.

						Suggests miR-155/TSPAN5 axis as a therapeutic target in DCA-resistant TNBC.
Kim et al., 2019 [62]	USA	Preclinical in vitro and in vivo experimental study	MDA-MB-231, MDA-MB-436, MDA-MB-468, Hs578T, HCC1806, HCC1569, DU4475	LNEs encapsulating DAC and PAN targeted via LPAR1	Vehicle or free drug lacking the LNE-mediated targeting	LPAR1-targeted LNEs encapsulating DAC/PAN selectively killed CDH1-/FOXM1+ TNBC cells.<br><br>Tumor accumulation of LNEs was significantly higher in TNBC models.<br><br>Epigenetic reversal via demethylation (CDH1) and gene repression (FOXM1) was achieved.<br><br>Minimal toxicity to non-cancerous cells.<br><br>Combination therapy was more effective than monotherapy.
Cooper et al., 2012 [63]	USA	Preclinical in-vitro experimental study	MDA-MB-231, BT20	Romidepsin (HDAC inhibitor) + DAC (DNA	Monotherapy (DAC-only and Romidepsin-only)	Combination of romidepsin + DAC significantly:<br>Induced apoptosis (cleaved PARP,

				methyltransferase inhibitor)	DMSO vehicle control	caspase-3)<br>Re-expressed sFRP1 (up to 1302-fold in MDA231)<br>Decreased cell viability in TNBC<br><br>shRNA knockdown of sFRP1 abrogated therapeutic response.<br><br>Exogenous recombinant sFRP1 mimicked drug effects.
Fan et al., 2024 [64]	China	In vitro study with functional assays	BT-549, HCC1937, MDA-MB-231	Bifunctional inhibitor J208 targeting HDAC1 and DNMT1	Untreated cells<br><br>Cells treated with HDAC inhibitor vorinostat and DNMT inhibitor (alone or in combination)	J208 simultaneously inhibits HDAC1 and DNMT1.<br><br>Promotes apoptosis and G0/G1 arrest in TNBC cells.<br><br>Reduces migration/invasion (inhibits EMT).<br><br>Triggers innate immune response via viral mimicry.<br><br>Demonstrates synergism vs. separate HDAC/DNMT inhibitors.
Gao et al., 2022 [65]	China	Preclinical in vivo	4T1	P/D-mMSNs (PTX +	NS (normal	Synergistic effect of PTX/DAC-

		and in vitro experimental study using murine models and TNBC cell lines		DAC loaded macrophage-mimicking mesoporous silica nanoparticles), with or without anti-PD-1	saline)<br><br>PTX alone<br><br>DAC alone<br><br>aPD-1 alone	mMSNs with aPD-1:<br>Enhanced tumor accumulation due to macrophage membrane camouflage<br>Increased cellular uptake and co-delivery of PTX and DAC<br><br>Induction of immunogenic cell death evidenced by:<br>↑ CRT exposure (52.93%)<br>↑ ATP secretion (35.31 nmol/L)<br>↑ HMGB1 release (42.78 ng/mL)<br><br>Reversal of T cell exhaustion:<br>↑ $CD8^+$ CTLs<br>↓ Tregs<br>↑ IFN-γ and TNF-α levels<br><br>Memory immune response: 43.77% $CD8^+$ TEM in spleen, tumor rejection upon re-challenge
Butler et al., 2020 [75]	USA	Preclinical experimental study using in vitro cell	Parental: MDA-MB-231 ("231")	AZA	Vehicle	Brain-metastatic TNBC cells (231Br) are more aggressive than parental cells in vitro and in vivo.

		lines and an in vivo mouse model	Brain-metastatic: MDA-MB-231Br ("231Br")			AZA is more effective in 231Br cells:<br>Lower IC50<br>Greater apoptosis<br>Downregulation of anti-apoptotic BCL-2<br><br>AZA reduces tumor burden and prolongs survival in vivo:<br>Median survival: 50 days vs 42 days<br>Tumor burden (via BLI) significantly decreased<br><br>AZA acts via epigenetic modulation:<br>Demethylation of keratin 18<br>Inhibition of DNMT3a<br><br>AZA inhibits pro-metastatic signaling:<br>Wnt-3, Wnt-4, GSK-3, β-catenin were all reduced with AZA treatment<br>Cell migration and invasion suppressed

						Potential clinical implications:<br>Keratin 18 hypermethylation is proposed as a diagnostic or predictive biomarker.
Banerjee et al., 2023 [76]	UK	Preclinical in vivo and in vitro experimental study	4T1	PDT using verteporfin (liposomal Visudyne™) activated by laser<br><br>DAC	Tumor-bearing but untreated; received saline only	Combination therapy resulted in:<br>Enhanced tumor necrosis and apoptosis.<br>Increased CD4+/CD8+ T-cell populations.<br>Upregulation of anti-tumor immune genes in breast and spleen.<br>Reversal of metastasis (reduced to 4% vs. 40% in control).<br>Evidence that immune surveillance was reactivated.<br><br>Strong correlation between molecular (gene/protein) data and histological findings confirmed anti-tumor immunity.
Nakajima et al., 2022 [66]	Japan	Preclinical in vitro study	D-type: MDA-MB-468, HCC38<br><br>G-type: MDA-MB-453,	DAC	Vehicle	Classification of TNBC lines into D-, G-, and R-types based on response to decitabine:<br>D-type: Apoptosis induction

			MDA-MB-157, MDA-MB-231, HCC1143<br><br>R-type: Hs578T, HCC1187, HCC1937			G-type: Cell cycle arrest via p21<br>R-type: Resistance to decitabine, sensitivity to cisplatin<br><br>DAC enhances cisplatin cytotoxicity, especially in D- and G-type cells.<br><br>NOXA plays a central role in mediating cytotoxicity; its expression is epigenetically suppressed and reactivated by decitabine.<br><br>Combination therapy (DAC + Cisplatin) showed synergistic effects, especially in partially resistant G-type lines.
Kong et al., 2015 [67]	China	Laboratory-based experimental study + retrospective analysis of clinical tissue samples	MDA-MB-231, MDA-MB-435, HCC-1937	Treatment of MDA-MB-231 cells with demethylating agent DAC; also siRNA-BRMS1 knockdown	No treatment or negative control siRNA	BRMS1 is frequently down-regulated in TNBC tissues and cell lines (esp. MDA-MB-231).<br><br>Hypermethylation of BRMS1 promoter is common (53.4% of TNBC tissues vs 24.1% of normal

						tissues; P<0.001).<br><br>BRMS1 methylation correlates significantly with larger tumor size and higher TNM stage.<br><br>Demethylation with 5-Aza-dC restores BRMS1 expression and reduces invasive capacity in vitro.<br><br>siRNA-mediated BRMS1 knockdown increases invasion.<br><br>BRMS1 inactivation may play a role in metastasis of TNBC.<br><br>Suggests BRMS1 as potential biomarker and therapeutic target.
Miyagawa et al., 2018 [77]	Japan	Laboratory experimental study on cell lines and human TNBC tissues (translational research; not a	HCC1937, BT-20, HCC70, MDA-MB-231, BT-54	DAC; siRNA-mediated knockdown; LRRC26 overexpression plasmid	Vehicle or control plasmid/siRNA	LRRC26 expression is frequently and specifically downregulated in TNBC due to promoter CpG island hypermethylation.<br><br>LRRC26 downregulation is significantly associated with higher

		clinical trial)				histological grade (more aggressive tumors).<br><br>Restoration of LRRC26 expression (by demethylation or overexpression) suppresses:<br>Anchorage-independent growth (soft agar assay).<br>Migration and invasion (transwell and IncuCyte assays).
Wang et al., 2020 [78]	China	In vitro and in vivo preclinical study	MDA-MB-231, BT549, MDA-MB-468, MDA-MB-453	DAC	pEGFP-C1 vector (empty plasmid)	WDR41 inhibits the AKT/GSK-3β/β-catenin pathway:<br>Overexpression: ↓p-AKT ser473 by 75% (P = .0009), ↓p-GSK-3α/β by 30% (P = .0376)<br>↑Cleaved caspase-3 (3-fold), indicating apoptosis<br>↓β-catenin nuclear translocation<br><br>Combined treatment with 5-aza-dC enhanced these effects, suggesting epigenetic modulation of signaling.<br><br>Knockdown of WDR41 activated

						AKT/GSK-3β/β-catenin, increasing proliferation and migration.
Umeh-Garcia et al., 2022 [79]	USA	Experimental laboratory-based preclinical study (in vitro with in silico analyses)	BT549, HCC1937, MDA-MB-231	ADC– global demethylating agent<br><br>CRISPR-deadCas9 system: Tet1-dCas9 (demethylation) and VP64-dCas9 (transcriptional activation)	Vehicle	LRIG1 is epigenetically silenced in TNBC via CpG methylation at both the promoter and enhancer levels.<br><br>High LRIG1 methylation is linked with poor overall survival in ER-negative breast cancer patients.<br><br>Restoring LRIG1 expression via targeted CRISPR-dCas9 reprogramming reduces cancer cell viability more effectively than global demethylation.<br><br>Demethylation + transcriptional activation through the dCas9 system is specific, does not induce off-target expression, and provides a proof-of-concept for epigenetic reprogramming as therapy.
Al-dulaimi et al., 2024 [80]	UK	In vitro experimental study	HS578T	ADC<br><br>+/- Trichostatin A	Vehicle	Telomere length : 5-aza treatment led to significant telomere elongation in all tested breast cancer

				(TSA, a histone deacetylase inhibitor)		lines.<br><br>DNA damage : Detected via γ-H2AX immunofluorescence. 5-aza increased DSBs globally and at telomeres specifically.<br><br>Telomerase activity: Only marginally increased despite hTERT upregulation. Likely due to non-functional splice variants.<br><br>POLD3 expression :Strongly upregulated in all lines after 5-aza exposure. POLD3 is known to regulate telomere elongation via ALT.<br><br>TSA co-treatment : Did not enhance effects of AZA and sometimes reduced POLD3 expression, suggesting antagonism.
He et al., 2024 [68]	China	Preclinical in vivo and in vitro experimental study	4T1 and PTX-resistant 4T1/PTX	DAC/PTX NPs@αPD-L1 (nanoparticle co-	PBS, DEC+PTX (free drug), DEC/PTX NPs	Enhanced therapeutic effect: DAC/PTX NPs@αPD-L1 showed the strongest inhibition of tumor

				delivery of DAC and PTX with anti-PD-L1 antibody)	(without αPD-L1)	growth, metastasis, and longest survival in mice.<br><br>Mechanistic synergy: Combining decitabine (epigenetic modulator), PTX (chemotherapy), and αPD-L1 (immunotherapy) led to:<br>Reversal of EMT and CSC phenotypes<br>Increased ICD and CRT expression<br>Improved T cell-mediated immunity<br>Synergistic tumor suppression effects<br><br>Tumor targeting: Nanoparticles demonstrated sustained and targeted drug release at tumor sites via EPR and PD-L1 antibody guidance.
Xiong et al., 2022 [69]	China	Preclinical experimental study using in vitro cell lines and in vivo animal models	MDA-MB-231 and HCC1806 cell lines with DAB2IP modulation	Pretreatment with DAC followed by DOC	DOC only	DAB2IP is a tumor suppressor: Downregulation leads to increased chemoresistance and stemness in TNBC.<br><br>DAB2IP inhibits RAC1-mediated β-catenin nuclear transport, reducing

						stem-like traits and resistance.<br><br>Epigenetic silencing of DAB2IP via methylation is a mechanism of resistance in TNBC.<br><br>DAC treatment reverses methylation, restores DAB2IP, and enhances DOC response.<br><br>Combination therapy (DAC+DOC) shows synergistic anti-tumor effect, especially in resistant models.
Elango et al., 2021 [70]	Qatar	Experimental, in vitro study using TNBC cell lines and bioinformatics analyses	MDA-MB-231 and BT-549	DNA methyltransferase inhibitors (DNMTi) – Decitabine and 5-Azacytidine	Vehicle	Epigenetic therapy via DNMT inhibition reprograms transcriptional and non-coding RNA networks in TNBC:<br>Activates tumor suppressor pathways (TP53)<br>Suppresses oncogenic drivers (FOXM1, ERBB2, CREB1)<br>Induces apoptosis, necrosis, and G2/M arrest<br>Reduces colony formation ability via TP53-TPX2 axis

						lncRNAs play a central role in epigenetic modulation: Downregulation of MALAT1 is significant, as it's linked to chemoresistance and tumor aggressiveness.<br><br>Survival analysis: Several gene signatures impacted OS/DFS, e.g., high NUPR1, PIK3CB, ESSRA = worse OS; high FTH1 = better DFS
Vernier et al., 2020 [71]	Canada	Preclinical experimental study (in vitro and in vivo xenograft models)	MDA-MB-231, MDA-MB-436, MDA-MB-468	C29 (ERRα inhibitor)<br><br>5-azadC (5-aza-2′-deoxycytidine; decitabine; DNMT inhibitor)<br><br>Combination therapy (C29 + AZA)	Vehicle	There is a feedforward loop between DNMT1 and ERRα where: DNMT1 stabilizes ERRα.<br>ERRα promotes DNMT1 expression and methionine cycle enzymes.<br><br>Dual inhibition (C29 + AZA): Interrupts this loop.<br>Triggers promoter demethylation of IRF4.<br><br>Derepressed IRF4 exerts anti-proliferative effects in breast cancer.

						IRF4 expression correlates with favorable patient survival, validated in GSE3143 and GSE7390 cohorts.
Pacaud et al., 2025 [72]	USA	Preclinical in vitro and in vivo experimental study	MDA-MB-231 and SUM149PT	Combination of PARP inhibitor (Talazoparib) + DAC	Vehicle	Synergy between low-dose Talazoparib + DAC is significantly more potent in BRCA-mutant models than monotherapy.<br><br>Combination allows lower dosing of Talazoparib, potentially reducing clinical toxicity.<br><br>Enhanced DNA damage (↑ p-H2A.X), ↑ micronuclei, ↓ RAD51 foci in HRR-deficient cells.<br><br>SUM149PT (natural BRCA1 mutant) and engineered M231BRCA cells show strongest responses.<br><br>Minimal effects in HRR-proficient cells despite some anti-proliferative activity.

AKT – Protein Kinase B; BRCA – Breast Cancer Genes; CDH1 – E-Cadherin; CREB1 – cAMP Response Element Binding Protein 1; DAB2IP – Disabled Homolog 2 Interacting Protein; DNMT / DNMT1 – DNA Methyltransferase; DPN – Diarylpropionitrile; ERα – Estrogen Receptor Alpha; ERβ – Estrogen Receptor Beta; ERBB2 – Erb-B2 Receptor Tyrosine Kinase 2; ESSRA – Estrogen-Related Receptor Alpha; FOXM1 – Forkhead Box Protein M1; FTH1 – Ferritin Heavy Chain 1; GSK – Glycogen Synthase Kinase-3; HMGB1 – High-Mobility Group Box 1; HRR – Homologous Recombination Repair; hTERT – Human Telomerase Reverse Transcriptase; IGF-1 – Insulin-like Growth Factor 1; IKKb – Inhibitor of Nuclear Factor Kappa-B Kinase Subunit Beta; IRF4 – Interferon Regulatory Factor 4; LPAR1 – Lysophosphatidic Acid Receptor 1; LRRC26 – Leucine-Rich Repeat-Containing Protein 26; MYC – MYC Proto-Oncogene, bHLH Transcription Factor; NOXA – Phorbol-12-myristate-13-acetate-Induced Protein 1; NUPR1 – Nuclear Protein 1; PARP – Poly (ADP-ribose) Polymerase; PDT – Photodynamic Therapy; PIK3CB – Phosphoinositide 3-Kinase Catalytic Subunit Beta; POLD3 – Polymerase Delta 3; Rap1B – Ras-Related Protein 1b; RUNX3 – Runt-Related Transcription Factor 3; sFRP1 – Secreted Frizzled-Related Protein 1; STING – Stimulator of Interferon Genes; TP53 – Tumor Protein P53; TPX2 – Targeting Protein for Xklp2; TRAF6 – Tumor Necrosis Factor Receptor-Associated Factor 6; TSPAN5 – Tetraspanin 5; VEGF – Vascular Endothelial Growth Factor; AZA – Azacitidine; DAC / ADC – Decitabine / 5-aza-2'-deoxycytidine; DEX – Dexamethasone; DOC – Docetaxel; PAN – Panobinostat; PTX – Paclitaxel; ATP – Adenosine Triphosphate; CRT – C-Reactive Protein or Chemoradiotherapy; CSC – Cancer Stem Cells; DNA – Deoxyribonucleic Acid; DSBs – Double Strand Breaks; EMT – Epithelial Mesenchymal Transition; EPR – Enhanced Permeability and Retention; γ-H2AX – Phosphorylated Form of Histone H2AX; miR / miR-155 – microRNA / microRNA-155; lncRNA – Long Non-Coding RNA; shRNA – Short Hairpin RNA; CTLs – Cytotoxic T Lymphocytes; ICD – Immunogenic Cell Death; IFN-γ – Interferon Gamma; IFN-β – Interferon Beta; IFN-I – Interferon Type I; ISGs – Interferon-Stimulated Genes; MHC-I – Major Histocompatibility Complex Class I; M-MDSCs – Monocytic Myeloid-Derived Suppressor Cells; PD-1 – Programmed Cell Death Protein 1; PD-L1 – Programmed Cell Death Protein Ligand 1; Tregs – Regulatory T Cells; TEM – Effector Memory T Cells; ALT – Alternative Lengthening of Telomeres; DFS – Disease-Free Survival; LNEs – Lipid Nanoemulsions; mMSNs – Macrophage-Membrane-Camouflaged Mesoporous Silica Nanoparticles; NCT – Neoadjuvant Chemotherapy; OS – Overall Survival; pCR – Pathological Complete Response; sTILs / iTILs – Stromal / Intratumoral Tumor-Infiltrating Lymphocytes; TNBC – Triple-Negative Breast Cancer; CRISPR – Clustered Regularly Interspaced Short Palindromic Repeats; DMEM – Dulbecco's Modified Eagle Medium.
